# Transfer RNA derived fragment, tRF-Glu-CTC, aggravates the development of neovascular age-related macular degeneration

**DOI:** 10.7150/thno.92943

**Published:** 2024-01-27

**Authors:** Yu Liang, Lingjie Kong, Yuelu Zhang, Yihan Zhang, Mingsu Shi, Jiaqiu Huang, Hongyu Kong, Siyi Qi, Yunlong Yang, Jiaxu Hong, Meidong Zhu, Xiangjia Zhu, Xinghuai Sun, Shujie Zhang, Lianqun Wu, Chen Zhao

**Affiliations:** 1 Eye Institute and Department of Ophthalmology, Eye & ENT Hospital, Fudan University, 83 Fenyang Road, Shanghai, 200031, China.; 2 NHC Key Laboratory of Myopia (Fudan University); Key Laboratory of Myopia, Chinese Academy of Medical Sciences, 83 Fenyang Road, Shanghai, 200031, China.; 3 Shanghai Key Laboratory of Visual Impairment and Restoration, 83 Fenyang Road, Shanghai, 200031, China.; 4 Department of Cellular and Genetic Medicine, School of Basic Medical Sciences, Fudan University, Shanghai, 200032, China.; 5 Save Sight Institute, Discipline of Clinical Ophthalmology and Eye Health, University of Sydney, Camperdown, NSW 2000, Australia.; 6 New South Weals Tissue Bank, New South Weals Organ and Tissue Donation Service, Sydney Eye Hospital, 8 Macquarie Street, Sydney 2000, Australia.

**Keywords:** Age-related macular degeneration, choroidal neovascularization, tRNA-derived small noncoding RNAs, angiogenesis

## Abstract

**Rationale:** Angiogenesis expedites tissue impairment in many diseases, including age-related macular degeneration (AMD), a leading cause of irreversible blindness in elderly. A substantial proportion of neovascular AMD patients, characterized by aberrant choroidal neovascularization (CNV), exhibit poor responses or adverse reactions to anti-VEGF therapy. Herein, we aimed to unveil the function of newly identified transfer RNA-derived small RNA, tRF-Glu-CTC, in the pathology of CNV and determine its potential in inhibiting angiogenesis.

**Methods:** Small non-coding RNA sequencing and quantitative polymerase chain reaction were conducted to detect expression pattern of tRF-Glu-CTC in CNV development. Immunofluorescence staining, fundus fluorescein angiography and ex vivo choroidal sprouting assays were employed for the evaluation of tRF-Glu-CTC's function in CNV development. The role of tRF-Glu-CTC in endothelial cells were determined by in vitro endothelial cell proliferation, migration and tube formation assays. Transcriptome sequencing, dual-luciferase reporter assay and in vitro experiments were conducted to investigate downstream mechanism of tRF-Glu-CTC mediated pathology.

**Results:** tRF-Glu-CTC exhibited substantial up-regulation in AMD patients, laser-induced CNV model, and endothelial cells under hypoxia condition, which is a hallmark of CNV. Inhibiting tRF-Glu-CTC reduced angiogenesis and hypoxia stress in the neovascular region without neuroretina toxicity in laser-induced CNV model, showing an anti-angiogenic effect comparable to bevacizumab, while overexpression of tRF-Glu-CTC significantly augmented CNV. Mechanically, under hypoxia condition, angiogenin was involved in the production of tRF-Glu-CTC, which in turn triggered endothelial cell tubulogenesis, migration and promoted the secretion of inflammatory factors via the suppression of vasohibin 1 (VASH1). When downregulating VASH1 expression, the inhibition of tRF-Glu-CTC showed minimal suppression on angiogenesis.

**Conclusions:** This study demonstrated the important role of tRF-Glu-CTC in the progression of angiogenesis. Targeting of tRF-Glu-CTC may be an alternative to current anti-VEGF therapy for CNV in AMD and other conditions with angiogenesis.

## Introduction

Increasing human life expectancy has led to a growing global burden of age-related diseases, such as atherosclerosis, Alzheimer's disease, and age-related macular degeneration (AMD) 1. AMD is the leading sight-threatening diseases, affecting the vision of population over age 60 2. Vascular dysfunction is generally considered to be a major event in the late process of AMD 3,4, in which choroidal neovascularization (CNV) significantly disrupts the blood-eye barrier, leading to edema, hemorrhage, and retinal detachment, contributing to vision loss in most of the patients 5. Mounting evidence suggests that hypoxia, dysregulated pro-angiogenetic and inflammatory factors are major stimulators to promote endothelial cells migration and angiogenesis in CNV 6,7.

Currently, anti-VEGF therapies are widely accepted for the treatment of neovascular AMD 8. However, only approximate 30% of these patients achieve significant vision improvements after treatment 9. Limited efficacy and recurrence are common due to the persistent exudation, hemorrhage, and progressive fibrosis 9. In these stress conditions, a switch to a pro-inflammatory and myofibroblast-like phenotype of endothelial cells and pericytes represents a significant contributor that nullifies or attenuates the effect of anti-VEGF agents 10,11. In addition, indiscriminate VEGF inhibition impairs the protective effects of VEGF on neurons and retinal pigment epitheliums (RPE)^12^-^14^. Thus, it is of great perspective to investigate novel angiogenic agents as a target for restoring endothelial cell function and interfering with neovascular AMD with less resistance or side effects.

Epigenetic alteration is one of critical hallmarks of aging and age-related diseases 15. Transfer RNA-derived small RNAs (tsRNAs) is a broad spectrum of nucleotide fragments spliced from transfer RNAs (tRNAs) under stress conditions, including hypoxia, heat shock, and UV irradiation 16. They can be divided into two types, tRNA-derived fragments (tRFs, 14-30 nt) and tRNA halves (tiRNAs, 31-40 nt), according to their length and cleavage site 17. Nowadays, tsRNAs are known as the novel epigenetic regulators of cell differentiation, survival, and apoptosis in a tissue- and developmental-specific pattern 18-20. Angiogenin (ANG), as a well-documented angiogenesis-associated ribonuclease, is required for the generation of most tsRNAs 21. The critical role of ANG in pathological angiogenesis and neurodegeneration indicates that tsRNAs exhibit great potential to modulate these processes in a stress dependent manner 22,23. Dysregulated tsRNA expression has been reported in cardiovascular diseases, neural degeneration diseases, and cancers 24-26. However, the relationship between tsRNAs and neovascular AMD is still unclear.

Herein, we identified a tsRNA, tRNA-Glu-CTC (known as tRF-5030c), as a novel contributor involved in the development of neovascular AMD, which dramatically aggravated the choroidal angiogenesis through inhibition of vasohibin 1 (VASH1). Suppression of tRF-Glu-CTC alleviated CNV formation and ameliorated reginal hypoxia without detectable side effects or retinal toxicity. Taken together, this study revealed the role of tsRNA in pathological angiogenesis and provided a novel tsRNA-based strategy for the treatment of neovascular AMD.

## Methods

### Cell culture and treatment

Primary human umbilical vein endothelial cells (HUVECs) (purchased from ScienCell, USA) were cultured in ECM medium containing 5% fetal bovine serum (ScienCell, 1001) at 37°C and 5% CO_2_ chamber. ARPE-19 (purchased from ATCC, USA, CRL-2302) were cultured in DMEM medium containing 10% FBS at 37°C and 5% CO_2_ chamber. For hypoxia induction, they were incubated in hypoxia chamber with 1% O_2_, 5% CO_2_ and 94% N_2_ for 24 h. For VEGF stimulation assay, they were treated with 10 ng/ml and 50 ng/ml recombinant human VEGF165 protein (PeproTech, USA, 100-2) for 24 h.

RNA mimics and inhibitors were designed and produced to regulate the level of tRF-Glu-CTC. Lipofectamine RNAiMAX (Invitrogen, USA, 13778075) was used to transfer RNA oligonucleotides into cells, according to manufacturer's protocols. All oligonucleotides' sequences were designed and synthesized by Ribobio (Guangzhou, China), as shown in Table S1.

### Nuclear and cytoplasmic extraction of HUVECs

To extract the nuclear and cytoplasmic fractions, NE-PER Nuclear and Cytoplasmic Extraction Reagents (Thermo Fisher Scientific, USA, 78835) were used following the manufacturer's protocols. About 1 × 10^6^ HUVECs were harvested and washed with cold PBS buffer. After centrifugation (500 × g), the cell pellet was incubated sequentially with cytoplasmic extraction reagent I, a cytoplasmic extraction reagent II and nuclear extraction reagent to isolate the cytoplasmic and nuclear fractions. The extracts were further used to detect the levels of tRF-Glu-CTC using quantitative real-time polymerase chain reaction (RT-qPCR).

### Animals and laser induced CNV model

Wild-type C57BL/6J mice (6-8 weeks old, 20-25 g, male) were purchased from Gempharmatech (Jiangsu, China) and fed in air-conditioned room with 12-hour light-dark cycle. We created a laser-induced CNV model to mimic the hallmarks of neovascular AMD in mice 27. In detail, The mice were anesthetized by intraperitoneal injection of 1.25% avertin (Nanjing Aibei Biotechnology, China, M2910) and the pupil was dilated by tropicamide (Santen Pharmaceutical, Japan). A 532 nm laser (VITRA, Quantel medical, France, CS 40015) with the power of 110 mW and duration of 100 ms was used to make laser spots in each eye of mice. Every spot was 2-3 papillary diameters away from optic disc and the clear bubble formation indicated the disruption of Bruch's membrane. After laser administration, the mice were intravitreally injected with 1 μl tRF-Glu-CTC agomir/antagomir (200 μM), the equivalent amount of scramble control oligonucleotides or bevacizumab (30 μg/μl). Seven days after treatment, the mice were sacrificed by cervical dislocation and the eyes were enucleated to make flat-mounted choroids. Vasculature in flat mount was labeled with fluorescein coagulated Isolectin B4 (Sigma-Aldrich, USA, L2895, 1mg/ml, 1:50). The CNV vessels in lesion sites were analyzed using fluorescence microscope and ImageJ 2.0 software. The animal study was approved by the Animal Care and Use Committee of Eye & Ear Nose and Throat (ENT) Hospital Affiliated to Fudan University.

### Clinical specimens

This study was approved by the Ethical Committee of Eye & ENT Hospital Affiliated to Fudan University (2023-YS-084). Fourteen patients with neovascular AMD were recruited as wet AMD group, and 11 age-related cataract (ARC) patients served as control group (Table S2). The aqueous humour (AH) samples were collected prior to intravitreal anti-VEGF therapy (for AMD group) or prior to phacoemulsification surgery (for control group) in accordance with the Declaration of Helsinki, and the informed consents were obtained from all patients before inclusion. The exclusion criteria involved: (1) history of ocular diseases other than ARC or AMD; (2) history of intraocular surgery or ocular trauma; (3) history of intravitreal anti-VEGF treatment or photodynamic therapy within the last six months in the study eye or within the last three months in the fellow eye; (4) systemic inflammatory or autoimmune diseases, or receiving steroids or immunosuppressive drugs within half a year before the study; (5) recent vascular or cerebral events within the last six months; (6) Alzheimer's disease or other age-related neural degenerative diseases.

### Fundus fluorescein angiography

To quantify vascular leakage, fundus fluorescein angiography was performed 7 days after CNV formation. In detail, the mice were firstly anesthetized and pupils were dilated. Then, mice were intraperitoneally injected with 100 μl 5% fluorescein sodium (Alcon Laboratories, USA). After 5 minutes, fundus angiogram photos with hyperfluorescence or leakage within the lesion area were acquired using OPTO-RIS system (Optoprobe, England). ImageJ 2.0 software were used to calculate the area of vascular leakage of each CNV lesion.

### Electroretinography (ERG)

ERG measurement was performed 1 week and 4 weeks after intravitreal injection. Before the measurement, mice were maintained in a dark room over 12 hours and were anesthetized under dim red light. Pupils were dilated and ERG was recorded in both eyes of mice via Espion Electrophysiology System (Diagnosys LLC, USA). A series of stimulus intensities (0.01, 0.1, 1.0, 3.0, 10.0, 30.0 cd s/m^2^) was applied for dark-adapted ERGs. The amplitude of the a-wave was calculated from the baseline to the maximum peak of a-wave. The amplitude of the b-wave was calculated from the maximum peak of a-wave to the maximum peak of b-wave.

### Optical coherence tomography (OCT)

To evaluate the changes of retinal structure, ISOCT system (Optoprobe) was used to capture OCT images of mice 4 weeks after weekly intravitreal treatment. Vertical OCT scan involved the optic disk of each eye was selected. The total retinal thickness and thickness of isos-rpe layer were measured using octSegmentation software. Total retinal thickness was calculated from inner limiting membrane to RPE. The thickness of isos-rpe was calculated from photoreceptor inner segment/outer segment junction layer to RPE.

### Choroidal sprouting assay

Six-week-old mice were sacrificed and the eyes were kept in DMEM medium (Gibco, USA) on ice before dissection. Then, 1 × 1 mm^2^ choroidal explants with RPE/choroidal/sclera complex were isolated and placed in the middle of growth factor-reduced Matrigel (BD Biosciences, USA, 354230) in a 24-well plate. 500 μl DMEM medium with 10% FBS (Gibco) or culture medium from transfected HUVECs was added. The plate was incubated at 37°C for 6 days. Images were taken on day 4 and day 6, and the sprouting area was measured with ImageJ 2.0.

### tiRNA & tRF sequencing

RNA samples were extracted from the RPE-choroid-sclera complexes of 3 laser-induced CNV model mice and 3 untreated mice on day 3 and day 7, respectively. After checking the integrity and quantity of RNA samples, we pretreated the tRNA derived fragments by performing 3'-aminoacyl deacylation and 3'-cP (2',3' -cyclic phosphate) removal for 3'adaptor ligation, 5'-OH (hydroxyl group) phosphorylation for 5'-adaptor ligation, m1A and m3C demethylation for efficient reverse transcription. Total RNAs were then sequentially ligated to 3' and 5' small RNA adapters, reversely transcribed, and amplified. 134-160 bp amplified fragments were extracted from PAGE gel and the libraries were quantified by Agilent 2100 Bioanalyzer (Agilent Technologies, USA). The denatured and diluted libraries were loaded onto reagent cartridge and forwarded to sequencing run on the Illumina NextSeq 500 system using the NextSeq 500/550 V2 kit (Illumina, USA, FC-404-2005) according to the manufacturer's instructions. For further analysis, the reads with 5'and 3'-adaptor bases trimmed were aligned allowing for 1 mismatch to the mature tRNA sequences or precursor tRNA sequences. The tRFs & tiRNAs expression profiles and differentially expressed small RNAs were identified based on the alignment results.

In tRFs & tiRNAs identification, small RNAs were categorized into five groups, tRF-5, tRF-3, tRF-1, tRF-2 and tiRNA. The tRF-5 and -3 were separately generated from 5' and 3' ends of the mature tRNA. The tiRNA were generated by specific cleavage in the anticodon loops of tRNA, in which tiRNA-5 start from 5' end of mature tRNA whereas tiRNA-3 start from the anticodon loop and end at 3' end.

The sequencing data have been uploaded in the NCBI Gene Expression Omnibus database (GSE210099).

### Cell sample collection and RNA sequencing

HUVECs were transfected with tRF-Glu-CTC mimic or Scr mimic (n = 4) using lipofectamine RNAiMAX according to the manufacturer's instructions. After 48 h, total RNAs of HUVECs were extracted using the TRIzol reagent. RNA integrity was evaluated using the Agilent 2100 Bioanalyzer (Agilent Technologies). The libraries were constructed using TruSeq Stranded mRNA Library Prep Kit (Illumina, RS-122-2101) according to the manufacturer's instructions. The sequencing was performed on an Illumina HiSeq X Ten platform and 150 bp paired-end reads were generated. About 50 M raw reads were generated for each sample. After being processed by Trimmomatic 28 and removing the low-quality reads, clean reads were collected to map to the human genome (GRCh38) 29. FPKM of each gene was calculated. Significantly differentially expressed genes (DEGs) were screened out based on the threshold of *P* value < 0.05 and fold change > 2 or fold change < 0.5. Gene otology (GO) and Kyoto encyclopedia of genes and genomes (KEGG) enrichment analysis of DEGs was performed to demonstrate the functions in different groups. Raw sequencing data have been uploaded to NCBI Sequence Read Archive (https://www.ncbi.nlm.nih.gov/bioproject/PRJNA862488).

### Cell proliferation assay

To detect cell proliferation ability, EdU Apollo567 In Vitro kit (RiboBio, C10310-1) was used. After treatment, HUVECs were incubated with 50 μM EdU for 2 h. Cells were fixed using 4% paraformaldehyde (PFA) and stained with dye solution according to manufacturer's protocol. DAPI (Thermo Fisher Scientific, 62248) was used for labeling cell nuclei.

### Tube formation assay

To assess the capillary-like structures, HUVECs (2 × 10^4^ per well) were plated into a 96-well plate with 50 μl Matrigel (BD Biosciences, 354230) at the bottom. The tube networks were detected after 4 h and the total branch length was quantified using Angiogenesis Analyzer in ImageJ 2.0 30. Among the Angiogenesis Analyzer results, total length, total branches length and total segments length were selected to evaluate the tube-forming ability of endothelial cells. In medium transfer experiment, the medium of transfected cells was collected 48 h after transfection. After 1000 rpm centrifugation, the supernatant medium was filtered using 0.22 μm filter (Merck Millipore, USA, SLGPR33RB) and was further used to culture normal HUVECs for 24 h.

### Cell migration assay

For wound healing assay, HUVECs were seeded into Ibidi Culture-Inserts 2 Well (Ibidi, Germany, 80209) in 12-well plates at a density of 1 × 10^5^ /ml. The Inserts were removed after 24 h. Then, the cells were cultured in ECM medium without FBS for 24 h. Images of migrated cells were obtained at 0 h and 12 h. The healing areas at different time points were calculated using ImageJ 2.0.

For Transwell assay, 2 × 10^4^ HUVECs were seeded onto the upper chamber of the 24-well Millicell Hanging Cell Culture Insert (Merck Millipore, MCEP24H48), containing 500 μl ECM medium without FBS, while 600 μl ECM medium with 10% FBS were added in the bottom chamber. After 12 h, the migrated cells on the lower membrane surfaces were fixed, stained and observed using microscope. In medium transfer experiment, the medium of transfected cells was added to normal HUVECs for 24 h before the whole experiment. Number of migrated cells were counted using ImageJ 2.0.

### Dual-luciferase reporter assay

The luciferase activity was detected using dual-luciferase reporter assay system (Promega, USA, E1910). In detail, the predicted tRF-Glu-CTC targeting sites in 3'UTR of VASH1 and the mutant sequences were cloned into the reporter vector pmirGLO. The plasmids were transfected together with tRF-Glu-CTC mimics or negative control into HUVECs using lipofectamine 3000 (Invitrogen, L3000015). After 48 h, cells were harvested to detect the luciferase activity according to manufacturer's protocol. The value of luciferase was measured using Tecan Spark microplate reader (Tecan, Switzerland). The information of plasmids containing the predicted tRF-Glu-CTC targeting sites was shown in Figure S1.

### Immunofluorescence assay

HUVECs were fixed with 4% PFA after treatment, followed by the permeation and blocking using 0.3% Triton X-100 and 1% BSA solution. Then, they were incubated with the primary antibodies of CD31 (Abcam, USA, ab76533, 1:500) or vimentin (Abcam, ab92547, 1:1000) over night at 4°C and were further incubated with donkey anti-rabbit (Abcam, ab150075, 1:1000) or anti-mouse secondary antibody (Abcam, ab150105, 1:1000) for 1 h at the room temperature. Finally, cell nuclei were stained with DAPI (Thermo Scientific, 62248). The images were captured using microscope. The mean fluorescence intensity of images was measured using ImageJ 2.0.

For RPE-choroid flat mount staining, the enucleated eyes were fixed in 4% PFA for 40 min. The posterior eyecups were cut into flat mounts, and the optic nerves and retinas were removed. Incubation with the primary antibodies, including angiogenin (Proteintech, 18302-1-AP, 1:100), vimentin (Abcam, ab92547, 1:1000) or fluorescein coagulated Isolectin B4 (Sigma-Aldrich, L2895, 1mg/ml, 1:50) were performed. After that, flat mounts were treated with anti-rabbit secondary antibody (Abcam, ab150075, 1:1000). For hypoxia detection, firstly, the mice were intraperitoneally injected with pimonidazole hydrochloride (Hypoxyprobe, USA, HP7, 60 mg/kg), and then, they were sacrificed 1 h later for the preparation of RPE-choroid flat mounts. An Dylight 549 fluorophore (HP-Red549)-conjugated monoclonal antibody against pimonidazole (Hypoxyprobe, HP7, 1:200) was used to label the hypoxic area.

### RT-qPCR

Total RNAs were extracted from HUVECs and choroidal tissues using RNeasy Mini Kit (Qiagen, German, 74004). tsRNAs were extracted using miRNeasy Mini Kit (Qiagen, 217048). For tsRNA quantification, the RNA pretreatment kit (Arraystar, USA, AS-FS-005) and rtStar First-strand cDNA Synthesis kit (Arraystar, AS-FS-003-02) were used to create cDNA libraries. Total RNAs were reversely transcribed to cDNA using PrimeScript RT Master Mix (Takara, Japan, RR036A). Then, RT-qPCRs were conducted with TB Green Premix Ex Taq (Takara, RR420A) through ^ΔΔ^CT method. The expression levels of mRNAs were normalized to that of β-actin. For the detection of tRF-Glu-CTC, the Bulge-Loop miRNA qRT-PCR Starter Kit (Ribobio, C10211-2) was applied. The levels of tsRNAs were calculated relative to the internal control U6. Using DNA electrophoresis, the sequence length of stem-loop qRT-PCR products were validated by comparison with that from standard tRF-Glu-CTC sample (Figure S1F). All primers for the detected genes were listed in Table S3. The stem-loop primers for tRF-Glu-CTC and U6 were designed and synthesized by Ribobio, China.

### Statistical analysis

All results were expressed as means ± SEM, which were obtained from at least 3 independent experiments. The statistical analysis was performed using Student's *t* test, one-way ANOVA test followed by Tukey's test or two-way ANOVA test in Prism 9 software (GraphPad Software, LLC., USA). A *P* < 0.05 was considered statistically significant.

## Results

### Characteristics of tsRNA expression in CNV tissue

**Figure 1:**
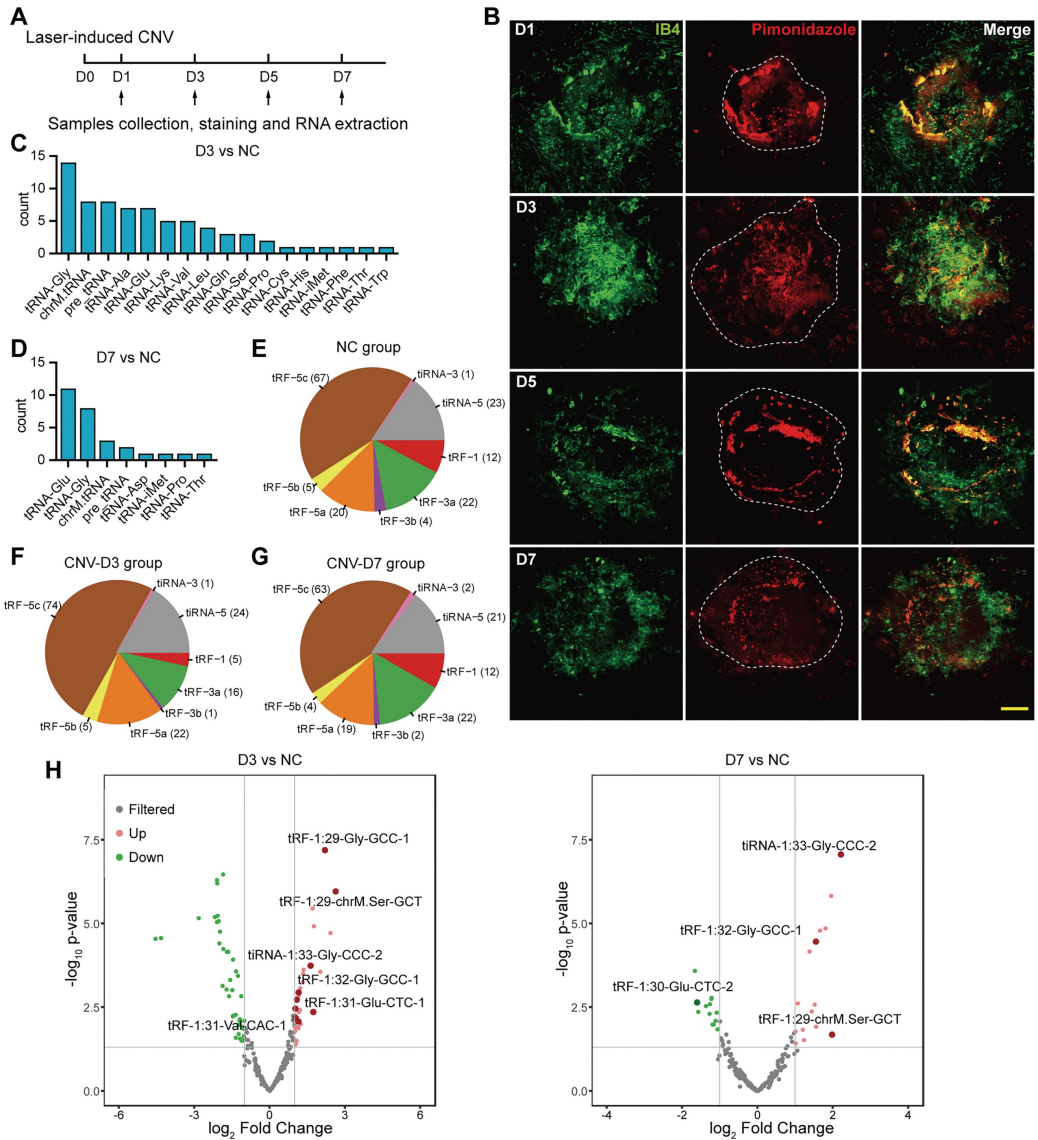
** The characteristics of transfer RNA-derived small RNAs (tsRNAs) in choroidal neovascularization (CNV) model.** (**A**) Sample collection, staining and RNA extraction were performed on day 1, 3, 5, 7 after the establishment of the laser-induced CNV model. (**B**) The neovascularization area and reginal hypoxia at different time points were assessed during the development of CNV. (**C**) The distribution of the precursor tRNAs type of differentially expressed tsRNAs between CNV-D3 group and NC group. (**D**) The distribution of the precursor tRNAs type of differentially expressed tsRNAs between CNV-D7 group and NC group. (**E-G**) The subtype distribution of tsRNAs screened out from NC (**E**), D3 (**F**) and D7 (**G**) groups. (**H**) The volcano plot of differentially expressed tsRNAs. The marked tsRNAs were distinguished with a threshold of log2FC > 1 or < -1 and *P* value <= 0.05.

To simulate the hallmarks of neovascular AMD, laser was used to disrupt Bruch's membrane and induce choroidal neovascularization in mice. The sprouting vessels and regional hypoxia stress were assessed on choroidal flat mounts (Figure 1A-B). During the formation of CNV, hypoxia marker pimonidazole was significantly enriched in the neovascular region, representing an elevated hypoxia stress, which rendered endothelial cells more prone to pro-angiogenic stimulation upon laser damage.

To investigate the global tsRNA expression pattern at different stages of CNV, three RPE-choroid-sclera complexes of laser-induced CNV mouse model on day 3 (named as D3 group) and day 7 (named as D7 group) were collected for tRF/tiRNA sequencing, and three RPE-choroid-sclera samples without treatment were taken as the control, respectively. According to the sequencing results, most of the differentially expressed tsRNAs were spliced from tRNAs encoding Glycine (Gly) and Glutamine (Glu) (Figure 1C-D), while the majority of the detected tsRNAs were distributed around 30 nt in length and were identified as tRF-5c or tiRNA-5, which were derived from 5' end of mature tRNAs (Figure 1E-G). Differentially expressed tsRNAs were identified with a threshold of log2FC > 1 or < -1 and *P* value <= 0.05. Compared with control group, 31 upregulated tsRNAs and 41 downregulated tsRNAs were screened out in the D3 group, while 19 upregulated and 13 downregulated tsRNAs were screened out in the D7 group (Figure 1H). The cluster heatmap of these differentially expressed tsRNAs were showed in Figure S2A. Among them, the expressions of a cluster of tsRNAs from tRNA-Glu and tRNA-Gly significantly increased on day 3 after laser injury. Then, on day 7, the levels of tsRNAs from tRNA-Glu decreased, while the levels of several tsRNAs from tRNA-Gly remained elevated, suggesting their different potential roles in the development of CNV.

### tRF-Glu-CTC expression increased in CNV region and endothelial cells under pro-angiogenic stress

**Figure 2:**
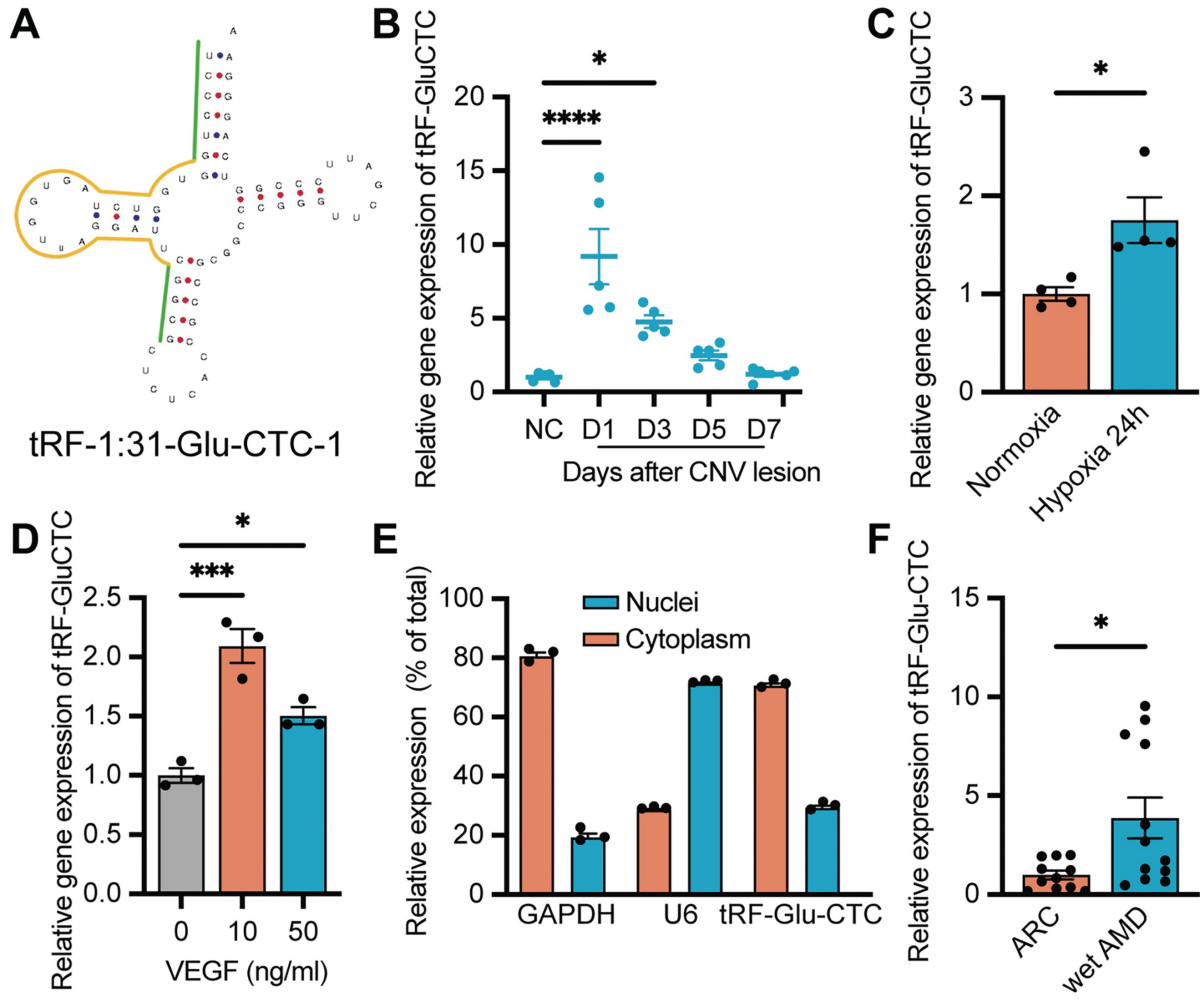
** Increased expression of tRF-Glu-CTC in CNV and endothelial cells under pro-angiogenic stress.** (**A**) The schematic diagram of the structure of tRF-Glu-CTC. (**B**) The expression levels of tRF-Glu-CTC at different time points after photocoagulation (n = 5, one-way ANOVA test). (**C**) The expression levels of tRF-Glu-CTC increased after being cultured in hypoxia condition for 24 h (n = 4). (**D**) The expression levels of tRF-Glu-CTC increased after the treatment of different concentration of VEGFA recombinant protein (n = 3, one-way ANOVA test). (**E**) The intracellular location of tRF-Glu-CTC in *human* umbilical vein endothelial cells (HUVECs). (**F**) The expression levels of tRF-Glu-CTC significantly increased in aqueous humour samples of wet age-related macular degeneration (AMD) patients compared to age-related cataract (ARC) controls. *P < 0.05, ***P < 0.001, ****P < 0.0001. All data were based on at least three independent experiments and were demonstrated as means ± SEM.

Based on the sequencing results, tRF-5c spliced from tRNA-Gly-GCC and tRNA-Glu-CTC accounted for the majority of all differentially expressed tsRNAs from tRNA-Glu and tRNA-Gly (Table 1). Based on the similarity between the sequence of tsRNAs originating from the same precursor tRNAs, we selected tRF-1:31-Glu-CTC-1 (named as tRF-Glu-CTC) and tRF-1:29-Gly-GCC-1 (named as tRF-Gly-GCC) as candidates to determine the expression pattern of these two tsRNA subfamily, in which the sequence of tRF-Glu-CTC was aligned with the sequence of tRNA-Glu-CTC-1-1 in human genome (HGNC symbol: TRE-CTC1-1) in the human tRF database, tRFdb 31 (Figure 2A). RT-qPCR assays revealed that tRF-Glu-CTC expression dramatically increased on day 1 and day 3, and gradually decreased thereafter (Figure 2B). In contrast, only on day 3 after CNV formation, a slight increase of tRF-Gly-GCC was detected (Figure S2B). Thus, tRF-Glu-CTC was selected for further studies.

Since a concurrent elevation of tRF-Glu-CTC was observed alongside the onset of CNV and an increase of local hypoxia stress, we further assessed the relationship between tRF-Glu-CTC and RPE-choroid complex in vitro. RPE and endothelial cells are two major participants in the development of CNV. Under pathological hypoxia stress, ARPE-19 exhibited a substantial elevation in HIF-1α and VEGFA levels, which were crucial processes in inducing CNV (Figure S2C-D). However, the expression of tRF-Glu-CTC in ARPE-19 did not show any significant changes (Figure S2E). On the other hand, in vitro study demonstrated a dramatic increase in tRF-Glu-CTC expression in primary cultured HUVECs under hypoxic conditions (Figure 2C). When treating with VEGFA, one of the most critical pro-angiogenetic factors in the pathological angiogenesis, an elevated level of tRF-Glu-CTC in HUVECs was detected as well (Figure 2D). The subcellular localization analysis revealed that tRF-Glu-CTC was mainly located in the cytoplasm (Figure 2E).

Furthermore, we collected the AH from AMD patients to confirm the clinical relevance of tRF-Glu-CTC with neovascular AMD. In AH samples from wet AMD patients with typical CNV lesions (n = 14), the level of tRF-Glu-CTC was significantly higher compared to AH from ARC patients without ocular comorbidities (n = 11) (Figure 2F), suggesting a potential correlation between the abnormal expression of tRF-Glu-CTC and neovascular AMD.

### tRF-Glu-CTC regulates CNV development in vivo

**Figure 3:**
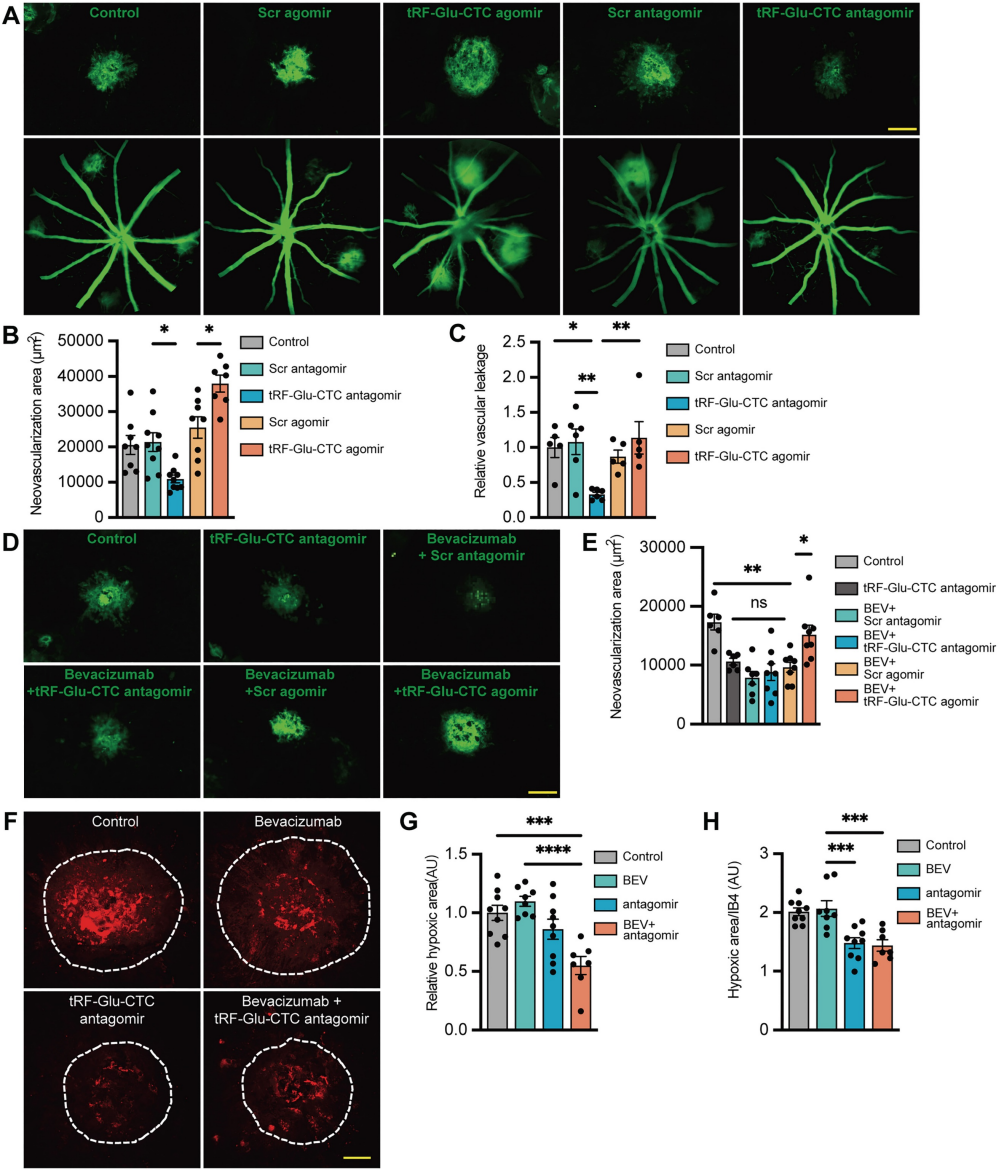
** tRF-Glu-CTC aggravates the development of CNV in vivo.** (**A**) The representative images of CNV lesion and vascular leakage 7 days after the treatment of tRF-Glu-CTC agomir or antagomir. Scale bar, 100 µm. (**B**) Quantitative analysis of the CNV area after tRF-Glu-CTC agomir/antagomir treatment revealed the promoting effect of tRF-Glu-CTC on neovascularization (n = 7-9, one-way ANOVA test). (**C**) Quantitative analysis of observed vascular leakage after tRF-Glu-CTC antagomir treatment (n = 5). (**D**) The representative images of CNV lesion after the combined treatment of bevacizumab and tRF-Glu-CTC antagomir. Scale bar, 100 µm. (**E**) Quantitative analysis of CNV area revealed tRF-Glu-CTC could diminish the outcome of anti-VEGF therapy, while antagomir exhibited similar outcomes to bevacizumab in inhibiting CNV (n = 6-8, one-way ANOVA test). (**F**) The representative confocal images of the hypoxic area obtained by pimonidazole staining after treatment of bevacizumab or tRF-Glu-CTC antagomir. Scale bar, 50 µm. (**G-H**) The hypoxic area (**G**) and the ratio of hypoxic area to IB4+ CNV area (**H**) of choroidal flat mounts at D4 demonstrated that tRF-Glu-CTC antagomir could decrease hypoxic stress in CNV region (n = 7-9, one-way ANOVA test). *P < 0.05, ** *P* < 0.01, ***P < 0.001, ****P < 0.0001, ns: not significant. All data were based on at least six independent experiments and were demonstrated as means ± SEM.

To determine the role of tRF-Glu-CTC in the development of neovascular AMD, tRF-Glu-CTC agomir and antagomir were promptly injected into the vitreous of laser-induced CNV mice following lesion formation. The increase of tRF-Glu-CTC after agomir administration was validated by RT-qPCR assays (Figure S2G). IB4 staining demonstrated that tRF-Glu-CTC overexpression could significantly aggravate choroidal neovascular lesions, whereas tRF-Glu-CTC inhibition by antagomir demonstrated the opposite effects, contributing to a 44% reduction in CNV area compared to control groups (Figure 3A-B). Consistent with these results, vascular leakage area in CNV region was reduced after the inhibition of tRF-Glu-CTC, but was not affected by its upregulation (Figure 3A and 3C).

The neovascular and hypoxic area surrounding CNV regions were further estimated to assess the inhibitory effects of tRF-Glu-CTC inhibition on CNV formation. Intravitreal administration of tRF-Glu-CTC antagomir, bevacizumab or a combination of the two agents all resulted in a ~50% reduction in the CNV area by day 7. However, the area of CNV exhibited no significant change compared to control when treated with bevacizumab in combination with tRF-Glu-CTC agomir (Figure 3D-E). Compared to bevacizumab, antagomir surprisingly attenuated regional hypoxia stress on day 3 after laser lesions, as evidenced by the reduction of pimonidazole+ hypoxic area and the ratio of hypoxic area to IB4+ CNV (Figure 3F-H). Furthermore, co-administration of tRF-Glu-CTC antagomir and bevacizumab resulted in a smaller hypoxic area in the CNV region compared to control or bevacizumab. (Figure 3F-H).

Then we conducted ERG and OCT analysis to assess the potential influence of tRF-Glu-CTC antagomir on neural function and retinal structure. Compared to PBS or bevacizumab, intravitreal injection of tRF-Glu-CTC antagomir in control mice did not affect the amplitude of the a-wave or b-wave in response to different flash stimuli after 1 week (Figure S3A), nor did tRF-Glu-CTC antagomir when it was injected weekly for 4 weeks (Figure S3B). However, bevacizumab slightly but significantly reduced the a-wave after 4-week repeated injections (Figure S3B). Besides, OCT images showed that repeated application of antagomir or bevacizumab did not reduce total retinal thickness or the isos-rpe thickness, which represented the structure of RPE layer (Figure S3C), suggesting that there was no detectable adverse effects of tRF-Glu-CTC antagomir on the retina structure. Collectively, these results indicated the great potential of tRF-Glu-CTC regulation in treatment of neovascular AMD.

### tRF-Glu-CTC plays a pro-angiogenic role in endothelial cells ex vivo and in vitro

**Figure 4:**
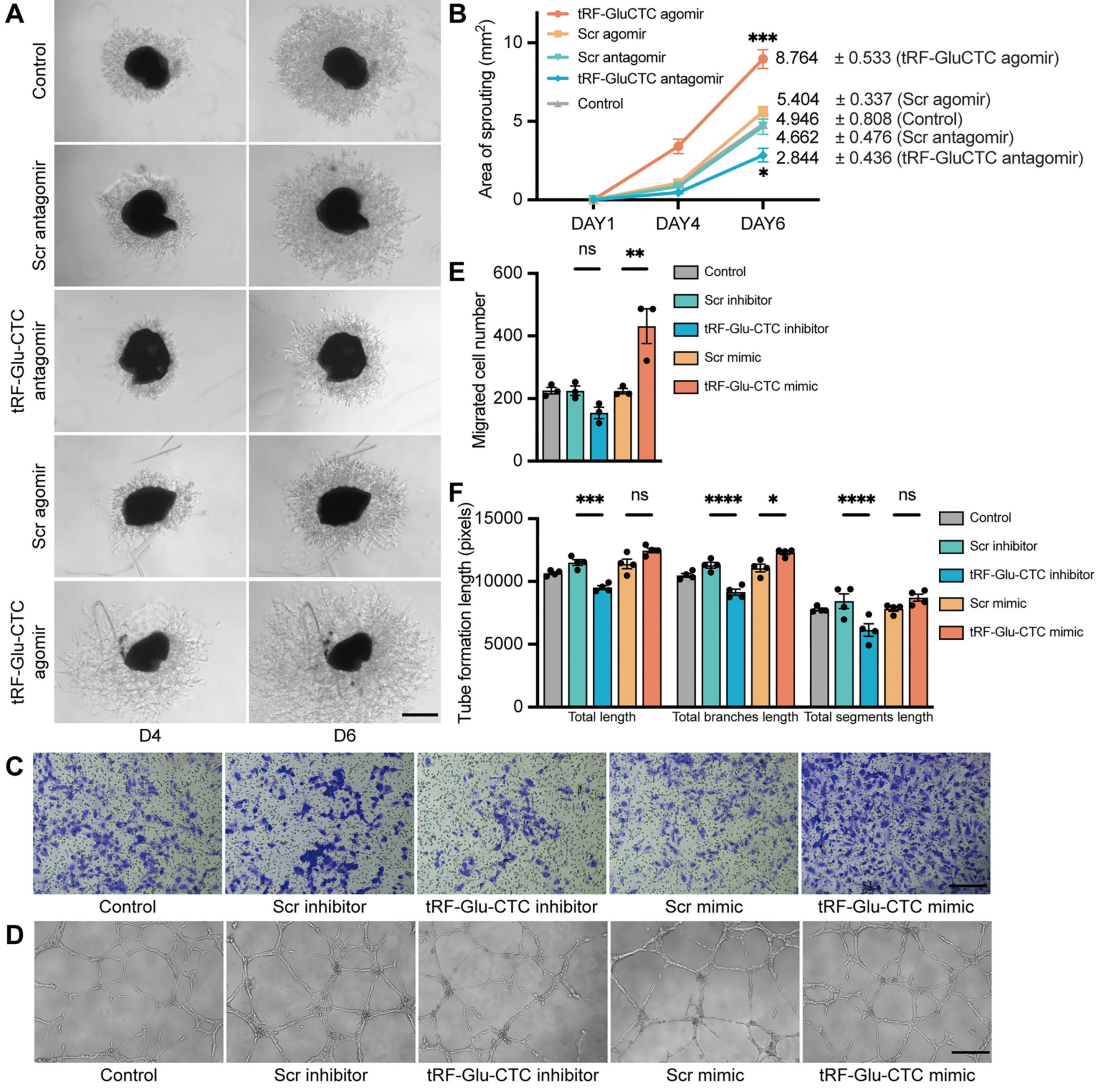
** tRF-Glu-CTC regulates the functions of endothelial cells ex vivo and in vitro.** (**A**) The representative images of choroidal sprouts at D4 and D6 after tRF-Glu-CTC agomir or antagomir treatment. Scale bar, 500 µm. (**B**) The sprouting areas at different time points indicated that tRF-Glu-CTC accelerated the angiogenic sprouting of choroidal vessels (n = 5, one-way ANOVA test). (**C-D**) The representative transwell images (**C**) and tube-forming images (**D**) of HUVECs transfected with tRF-Glu-CTC mimic or inhibitor. Scale bar, 250 µm. (**E-F**) Quantitative calculation of migrated cells (n = 3) (**E**) and tube formation length (n = 4) (**F**) showed that tRF-Glu-CTC promoted the migration and tube-forming ability of HUVECs. One-way ANOVA test was used for statistical analysis. *P < 0.05, **P < 0.01, ***P < 0.001, ****P < 0.0001, ns: not significant. All data were based on at least three independent experiments and were demonstrated as means ± SEM.

Next, an ex vivo choroidal sprouting assay was used to investigate the influence of tRF-Glu-CTC on choroidal vascular dysfunction. We examined the microvessel sprouting area of choroidal tissues isolated from mice 3 days after intravitreal injection of agomir or antagomir. tRF-Glu-CTC agomir dramatically stimulated the sprouting of choroidal vessels in choroid explants, while tRF-Glu-CTC antagomir considerably decreased sprouting area (Figure 4A-B), leading to impaired vascular network formation. Consistent with the ex vivo results, tRF-Glu-CTC mimic significantly promoted the migration and tube formation of HUVECs (Figure 4C-F, Figure S4A). The upregulation of tRF-Glu-CTC after mimic transfection was validated (Figure S2H). However, altered level of tRF-Glu-CTC did not affect the proliferative ability of HUVECs (Figure S4B). These data indicated the direct regulation of tRF-Glu-CTC on endothelial cell angiogenetic behavior.

### tRF-Glu-CTC was potentially modulated by hypoxia/ANG axis

**Figure 5:**
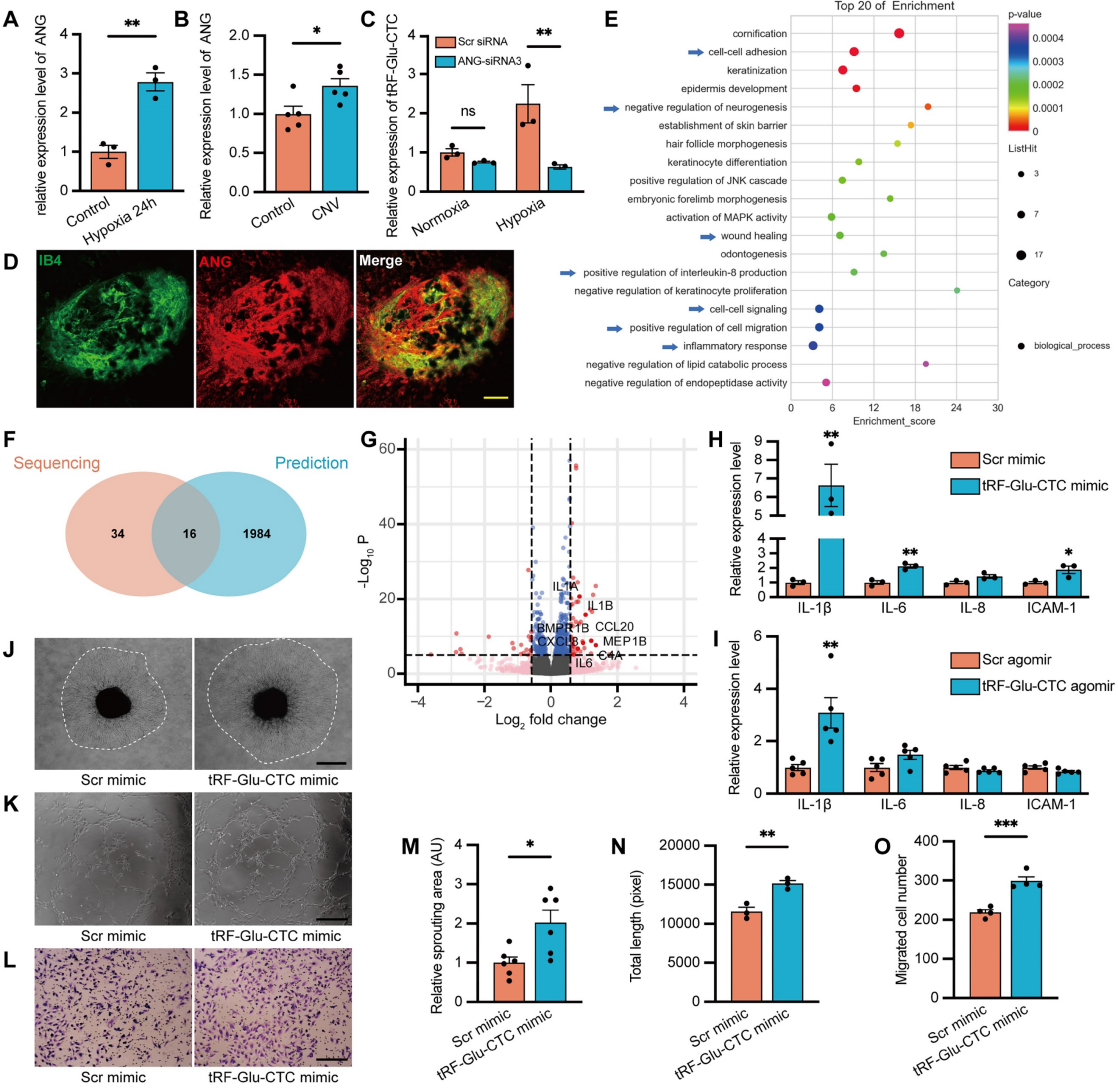
** The functional role of tRF-Glu-CTC is associated with angiogenin (ANG)-involved tsRNA generation and the secretion of inflammatory factors.** (**A**) The ANG expression was upregulated under hypoxia condition (n = 3). (**B**) The ANG expression was upregulated in RPE-choroid complex after CNV formation (n = 5). (**C**) The expression level of tRF-Glu-CTC decreased after the suppression of ANG in hypoxia condition, whereas the level revealed no difference in normoxia condition (n = 3, one-way ANOVA test). (**D**) Immunofluorescence staining revealed that ANG was colocalized with IB4+ neovascular region. Scale bar, 50µm. (**E**) The Gene Ontology (GO) functional enrichment analysis demonstrated the top 20 biological process (BP) terms enriched in the differentially expressed genes (DEGs). (**F**) Schematic diagram of the overlap between the top 50 enriched BPs of DEGs and enriched BPs of predicted target genes of tRF-Glu-CTC. (**G**) The volcano plot of DEGs based on a threshold of fold change > 1.5 or < 0.67 and *P* < 10^-5^. (**H & I**) The expression levels of related inflammatory factors significantly increased in HUVECs transfected with tRF-Glu-CTC mimic (n = 3) and RPE-choroid complex 4 d after CNV formation (n = 5). (**J & M**) The choroidal sprouting assay revealed that sprouting area was elevated by culture medium collected from tRF-Glu-CTC transfected HUVECs (n = 6). Scale bar, 500 µm. (**K & N**) The tube formation assay revealed that the total length and total branches length of HUVECs were reduced after 24 h culture with medium from tRF-Glu-CTC mimic transfected HUVECs (n = 3). Scale bar, 250 µm. (**L & O**) The transwell assay revealed that the migrated cells significantly increased when HUVECs transfected with tRF-Glu-CTC mimic were added to the bottom chamber (n = 4). Scale bar, 250 µm. *P < 0.05, **P < 0.01, ***P < 0.001. All data were based on at least three independent experiments and were demonstrated as means ± SEM.

With the initial observation of increased tRF-Glu-CTC expression and its pro-angiogenetic role, our subsequent focus was to investigate the underlying mechanism involving the generation of tRF-Glu-CTC. ANG plays a key role in angiogenesis and tsRNAs production 21. Consistent with the expression changes of tRF-Glu-CTC, the level of ANG was also significantly increased in HUVECs under hypoxia condition and in RPE-choroid complex after CNV formation (Figure 5A-B). Three siRNAs of ANG were designed to downregulate the expression of ANG, among which the siRNA with the highest interference efficiency was selected for further study (Figure S2F). ANG knockdown did not affect the level of tRF-Glu-CTC in HUVECs under normoxia condition, but obviously suppressed the expression of tRF-Glu-CTC under 24 h hypoxia treatment (Figure 5C). Moreover, in laser-induced CNV region, overexpressed ANG was majorly colocalized with the IB4+ area (Figure 5D), implying that ANG may play a role in the modulation of tRF-Glu-CTC in response to pathological stimulation, including hypoxic conditions.

### tRF-Glu-CTC regulates endothelial cell function via inducing the secretion of inflammatory factors

We next employed transcriptome sequencing to comprehensively assess global changes in gene expression following tRF-Glu-CTC upregulation, providing deeper insights into the functions of tRF-Glu-CTC in endothelial cells. Compared to controls, a total of 184 DEGs were screened out in HUVECs transfected with tRF-Glu-CTC mimic (Table S4), and their enriched function and pathway prediction were performed using GO and KEGG analysis (Table S5-6). The top 20 enriched biological processes were shown in Figure 5E. Next, the potential target genes of tRF-Glu-CTC were predicted based on targetScan and miRanda database (Table S7). Among the GO analysis of DEGs and predicted target genes (Table S8), a total of 16 enriched biological processes overlapped (Figure 5F). These processes were associated with inflammatory response, cell-cell adhesion, positive regulation of cell migration, and wound healing, suggesting that increased inflammation and migratory ability were two major characteristics of tRF-Glu-CTC upregulated endothelial cells.

Among the DEGs related to enriched biological processes, several genes were screened out as inflammatory cytokines (Figure 5G). RT-qPCR assays validated the increased expression of these factors, including IL-1β, IL-6, and ICAM-1 in HUVECs and RPE-choroid complex with elevated tRF-Glu-CTC (Figure 5H-I). In several disease model, these secreted factors contribute to an inflammatory microenvironment to exacerbate angiogenesis 32. We therefore collected the culture medium from tRF-Glu-CTC mimic-transfected HUVECs and found that the culture medium significantly promoted the angiogenetic sprouting of choroidal explants in ex vivo assay (Figure 5J and 5M). After 24 h of treatment with above culture medium, endothelial cells exhibited higher tube-forming and migratory ability, as shown by an increase in total length of tube formation assay (Figure 5K and 5N) and an increase in migrated cell numbers of Transwell assay (Figure 5L and 5O).

Vimentin, a biomarker of aging and age-related diseases, is upregulated in endothelial cells during the loss of normal function and the acquisition of a migratory mesenchymal phenotype 33. The upregulation of vimentin in endothelial cells is involved in the inflammatory response 34. In this study, immunofluorescence assays showed the increased expression of vimentin in tRF-Glu-CTC mimic-transfected endothelial cells with an enhanced migratory ability, while the expression of endothelial adhesion marker CD31 was not affected (Figure S5A-C). In vivo study showed that vimentin-expressing endothelial cells in choroid flat mounts accumulated around the CNV regions, suggesting their potential involvement in the vascular sprouting and CNV expansion (Figure S5D). Injection of tRF-Glu-CTC antagomir significantly reduced the vimentin+ area (Figure S5E-F).

Consequently, these results suggested that tRF-Glu-CTC mediated the inflammatory factors secretion and induced endothelial cells into a migratory phenotype, which facilitated pathological angiogenesis under stress condition.

### The function of tRF-Glu-CTC is partly mediated by suppression of VASH1

**Figure 6:**
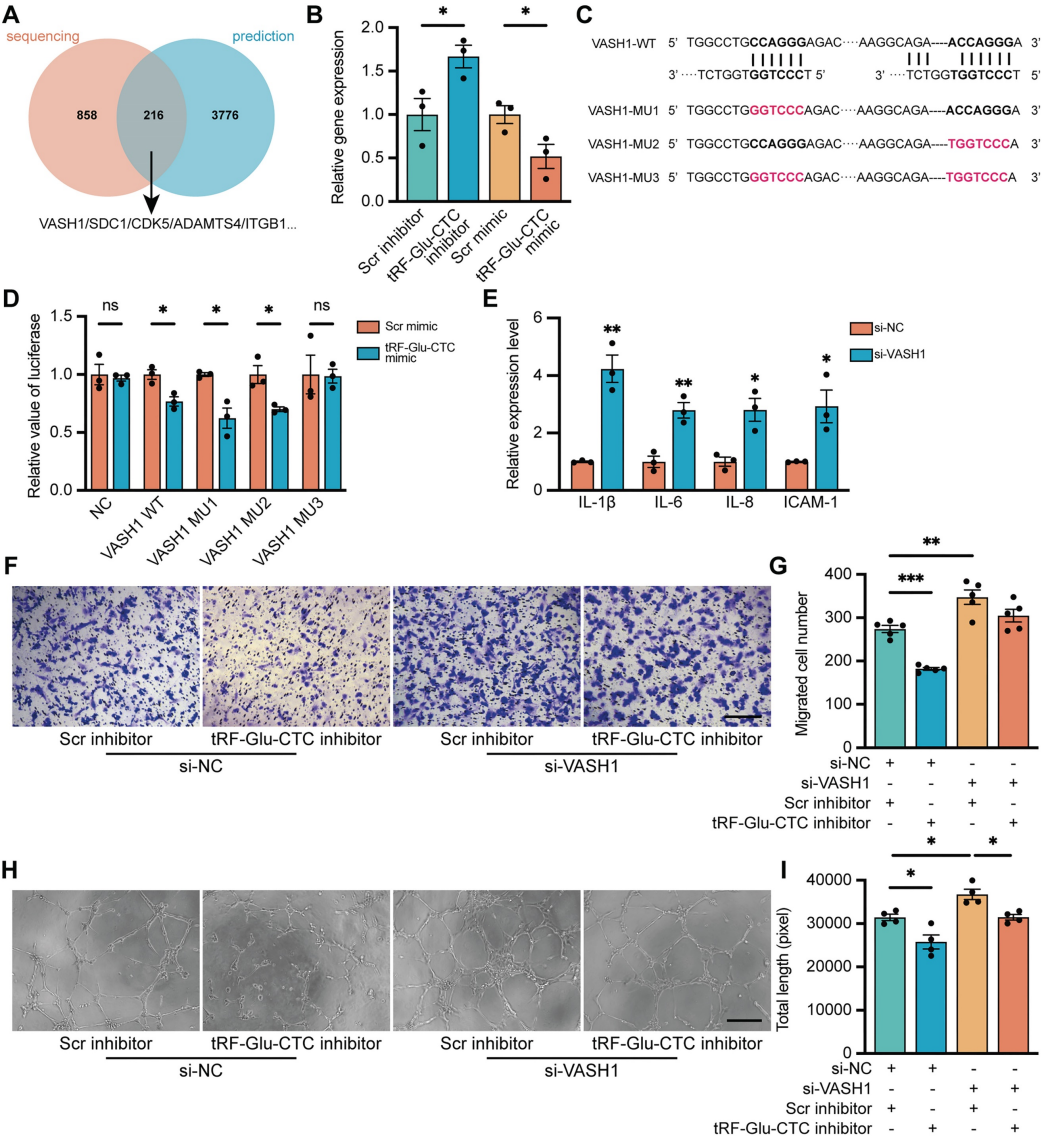
** Vasohibin 1 (VASH1) is involved in the function of tRF-Glu-CTC in the process of neovascularization.** (**A**) Schematic diagram of the target genes prediction, based on 3992 predicted genes from TargetScan and miRanda database and 1074 DEGs from sequencing results. (**B**) The expression level of VASH1 was suppressed by tRF-Glu-CTC mimic, whereas its level was elevated by tRF-Glu-CTC inhibitor (n = 3). (**C**) Schematic diagram of the predicted interaction sites and mutation sites between 3'UTR regions of VASH1 and tRF-Glu-CTC. (**D**) The measurement of luciferase activity validated that there was an interaction between 3'UTR region of VASH1 and tRF-GluCTC (n = 3). (**E**) The expression levels of related inflammatory factors significantly increased in HUVECs transfected with si-VASH1 (n = 3). (**F & G**) Transwell assay showed that VASH1 knockdown weakened the inhibitory effect of tRF-Glu-CTC inhibitor on the migration of HUVECs (n = 5, one-way ANOVA test). Scale bar, 250 µm. (**H & I**) Tube formation assay showed that tRF-Glu-CTC inhibitor suppressed the angiogenesis of HUVECs, while VASH1 knockdown reversed its effect (n = 4, one-way ANOVA test). Scale bar, 250 µm. *P < 0.05, **P < 0.01. ***P < 0.001, ns: not significant. All data were based on three independent experiments and were demonstrated as means ± SEM.

Given the similar structure of tsRNAs and miRNAs, the underlying molecular targets of tRF-Glu-CTC were predicted according to the principle of complementary base pairing. According to the overlap between all the significantly down-regulated genes and genes predicted by TargetScan and miRanda database, 216 candidate genes were screened out, of which 5 genes were selected for validation (Figure 6A). Among them, only the expression of VASH1 in HUVECs decreased in the presence of tRF-Glu-CTC mimic, and increased in the case of tRF-Glu-CTC inhibitor (Figure 6B, S6B), although a similar expression change was not observed in vivo (Figure S2J). Multiple evidences have shown the inhibitory effect of VASH1 on angiogenesis 35. In addition, VASH1 possesses detyrosinase activity that regulates the microtubule structure and cell migration, and its reduction may be implicated in acceleration of age-related vascular disease 36,37. There are five potential binding sites on 3'UTR region of VASH1 to tRF-Glu-CTC (Figure S6A). To further validate the target sites of tRF-Glu-CTC, VASH1 3'UTR sequences containing 2 adjacent target sites or corresponding mutant sites were added in the reporter vector pmirGLO for luciferase activity assay (Figure 6C). The results revealed that co-transfection of tRF-Glu-CTC mimic reduced the luciferase activity of pmirGLO-VASH1-WT, pmirGLO-VASH1-MU1, and pmirGLO-VASH1-MU2, but did not interfere the luciferase activity of pmirGLO-NC or pmirGLO-VASH1-MU3 (Figure 6D). These results indicated site 1 and 5 on 3'UTR of VASH1 were both the target sites of tRF-Glu-CTC.

### tRF-Glu-CTC/VASH1 axis is involved in the regulation of HUVECs

To determine the role of VASH1 in HUVECs, VASH1-siRNA was transfected into HUVECs to reduce its expression. The inhibitory effect of siRNAs was validated (Figure S2I). VASH1 knockdown increased the expression of vimentin (Figure S6E-F) and inflammatory factors (Figure 6E) in endothelial cells. Moreover, decrease of VASH1 accelerated the proliferation, migration, and tube formation of HUVECs (Figure 6F-I, Figure S6C-D). Transfection of VASH1-siRNA could rescue the inhibitory effects of tRF-Glu-CTC inhibitor on the migration and tube formation ability of HUVECs (Figure 6H-K). Collectively, these results showed that tRF-Glu-CTC modulated the functions and behaviors of endothelial cells via VASH1 inhibition.

## Discussion

Endothelial cell dysfunction and pathological vascular remodeling are the hallmarks of age-related diseases, where the overall ability of the organism is significantly compromised 38. Thus, targeting endothelial cells is a promising strategy against those diseases. Increasing studies on neovascular AMD have developed several modalities to restore sights by targeting angiogenesis 39,40. However, further understanding on its mechanism and exploration of new powerful therapeutic targets are still needed to bridge the shortcomings of current therapies 9. tsRNAs are involved in the pattern of aging and dysregulated angiogenesis, conferring a novel approach to prevent disease progression 41. In this study, the tsRNA expression pattern in CNV was investigated via tRF/tiRNA sequencing analysis. A tRNA derived fragment, tRF-Glu-CTC, was highly abundant in AH samples of wet AMD patients, as well as in CNV regions of mice and hypoxia-stimulated endothelial cells. tRF-Glu-CTC modulated endothelial cells behaviors by stimulating the secretion of pro-angiogenic factors and inhibiting the expression of VASH1, ultimately promoting neovascularization. Inhibition of tRF-Glu-CTC retarded the development of CNV, protected the retina from prolonged hypoxia stress without detectable structural and functional adverse effects, providing new insights for future treatment of AMD.

Angiogenesis is a complex multi-stage process consisting of extracellular matrix degradation, endothelial cell sprouting, migration, and maturation, while hypoxia and inflammation are two pivotal instigators 32,42. In laser induced CNV model, Burch's membrane and RPE rupture triggered inflammatory damage, evoking the penetrating of choroidal endothelial cells and subsequent angiogenesis 27. Our study and previous researches validated the increased inflammatory reaction and a substantial manifestation of hypoxia at the early stage of CNV formation 43, consistent with the significantly upward trend of tRF-Glu-CTC expression after laser injury. Hypoxia stress dramatically promoted the tRF-Glu-CTC production, which, in turn, amplified inflammation by promoting the inflammatory factors secretion. These evidences highlighted the interconnection between the endothelium expressed tRF-Glu-CTC, hypoxia and inflammatory stimulation during the occurrence of neovascular AMD.

However, given the complex microenvironments in outer retina, the role of tRF-Glu-CTC in other cells, including RPE and photoreceptor, are also require in future studies. Furthermore, the secretion of inflammatory factors makes it possible for tRF-Glu-CTC to propagate pro-angiogenetic phenotype to neighboring cells, supporting the significant contribution of tRF-Glu-CTC on CNV in a paracrine manner. Emerging evidence has shown that these inflammatory factors are important elements in senescence-associated secretory phenotypes, and identified vimentin as an age-related marker, which contribute to age-related inflammation, mitochondria dysfunction and pathological angiogenesis in age-related diseases 33,44. Thus, tRF-Glu-CTC may play a more significant role in the aging retina, where senescent cells are incompatible with vascular regeneration or maturation, but tend to accelerate angiogenesis by secreting these inflammatory factors 45. The impact of tRF-Glu-CTC in aging cells warrants thorough investigation.

It has now been identified that tsRNAs are not the random byproducts of tRNA metabolism under stress, but rather precisely produced to participate in the development of disease 16. ANG is a member of the ribonuclease superfamily that is expressed in the circulation and has the capacities to promote neovascularization 46. Under stress conditions, ANG has been proved to cleave tRNAs at anticodon loop and generate tiRNAs 47. It may explain why tRF-5c and tiRNA-5, which are spliced near the anti-codon region, account for the majority of the upregulated tsRNAs after laser lesion. Emerging studies exhibit that the tRNA cleavage function of ANG is associated with its subcellular location in response to cell stress 48. Under normal condition, ANG remains inactive in nucleus and is mainly bound to RNH1. Disruption of cellular homeostasis leads to ANG translocation and the activation of its ribonuclease functions. This can partly explain why ANG influences the expression of tRF-Glu-CTC under hypoxia condition rather than normoxia condition. In addition, the decrease in tRF-Glu-CTC in the later phase of the CNV model might be partially resulted from the decrease of aberrant stimulation and the redistribution of ANG. Further studies are needed to investigate the upstream mechanism involved in tRF-Glu-CTC production.

Mechanistically, it was found that tRF-Glu-CTC aggravated the development of CNV by suppressing the expression of VASH1 in a miRNA-like manner. VASH1 is the first identified tubulin detyrosinase that participates in mitosis, cardiac cell function, and trafficking in neurons by inducing post-translational modifications 36. As a typical angiogenetic inhibitor, VASH1 is preferentially expressed in endothelium behind the sprouting front to terminate angiogenesis 49. For example, the endocytosis and trafficking of VEGFR2 can be inhibited by VASH1 via its modulation of microtubules, resulting in reduced migration of endothelial cells and compromised angiogenesis 50. Besides, VASH1 is responsible for maintaining homeostasis in endothelial structure and function, and its down-regulation dramatically decreases stress tolerance of vasculature and even promotes stress-induced senescence 51. These functions of VASH1 make it a potential target to inhibit neovascularization and even attenuate inflammatory response in many diseases 52,53. In line with these findings, our study demonstrated that tRF-Glu-CTC-mediated VASH1 inhibition hindered the protective effects of VASH1 on vasculature and enhanced pro-angiogenetic stimulation by releasing inflammatory factors. These results, in combination with previous studies about VASH1, highlighted the importance of tRF-Glu-CTC/VASH1 axis in the development of neovascular AMD. While the in vitro experiments revealed an impact of tRF-Glu-CTC on the expression of VASH1, the in vivo experiments did not exhibit a similar pattern. This discrepancy between the in vitro and in vivo results suggested that the heterogeneity within the tissue and potential regulation of the microenvironment may influence the expression of VASH1 under tRF-Glu-CTC intervention. Besides, the inhibition of VASH1 promoted endothelial cell proliferation while tRF-Glu-CTC showed no influence on it, further implying the presence of other regulatory mechanisms between tRF-Glu-CTC and angiogenetic factors. Up to now, predicting downstream targets and underlying mechanisms of tsRNAs remains challenging. Unlike miRNAs that mainly interact with argonaute 2 and form miRNA-induced silencing complex, tsRNAs can perform non-canonical RNAi functions by binding to argonaute 1/3/4 19,54. The sequences of tsRNAs that aligned to target genes can be not only the miRNA-like 5' seed regions 55. Furthermore, researchers showed that the binding between tsRNAs and mRNAs encoding ribosomal proteins could enhance their translation and promote ribosome biogenesis 56. tsRNAs could be associated with RNA-binding proteins, including Y box binding protein 1 to directly regulate cell behaviors 26. Therefore, the molecular mechanism underlying tRF-Glu-CTC-induced angiogenesis requires deeper investigation.

## Conclusions

Several compelling evidence demonstrates that a novel tsRNA, tRF-Glu-CTC, contributes to the development of neovascular AMD by releasing inflammatory factors and inhibiting VASH1 to induce angiogenic phenotypes in endothelial cells. Inhibition of tRF-Glu-CTC should be further investigated as a potential target for the treatment of AMD and other angiogenesis-related diseases.

## Supplementary Material

Supplementary figures and tables.

## Figures and Tables

**Table 1:** Significantly upregulated transfer RNA-derived small RNAs derived from tRNA-Gly or tRNA-Glu on day 3 after choroidal neovascularization formation.

tRF_ID	Sequence	Type	Length	Precursor	Fold Change
tRF-1:16-Gly-TCC-1	GCGTTGGTGGTATAGT	tRF-5a	16	tRNA-Gly-TCC	5.37589146
tRF-1:29-Gly-GCC-1	GCATGGGTGGTTCAGTGGTAGAATTCTCG	tRF-5c	29	tRNA-Gly-GCC	4.62051055
tRF-1:32-Gly-CCC-2	GCATTGGTAGTTCAATGGTAGAATTCTCGCCT	tRF-5c	32	tRNA-Gly-CCC	3.404935
tiRNA-1:33-Gly-CCC-2	GCATTGGTAGTTCAATGGTAGAATTCTCGCCTC	tiRNA-5	33	tRNA-Gly-CCC	3.10909854
tRF-1:32-Glu-TTC-3	TCCCTGGTGGTCTAGTGGCTAGGATTCGGCGC	tRF-5c	32	tRNA-Glu-TTC	2.56384441
tRF-1:32-Glu-CTC-2	TCCCTGGTGGTCTAGTGGTTAGGATTTGGCGC	tRF-5c	32	tRNA-Glu-CTC	2.51036932
tRF-1:24-Gly-GCC-2-M3	GCATTGGTGGTTCAGTGGTAGAAT	tRF-5b	24	tRNA-Gly-GCC	2.36596196
tRF-1:32-Glu-CTC-1	TCCCTGGTGGTCTAGTGGTTAGGATTCGGCGC	tRF-5c	32	tRNA-Glu-CTC	2.33247089
tRF-1:30-Gly-GCC-1	GCATGGGTGGTTCAGTGGTAGAATTCTCGC	tRF-5c	30	tRNA-Gly-GCC	2.24459304
tRF-1:32-Gly-GCC-1	GCATGGGTGGTTCAGTGGTAGAATTCTCGCCT	tRF-5c	32	tRNA-Gly-GCC	2.23451271
tRF-1:29-Glu-CTC-1	TCCCTGGTGGTCTAGTGGTTAGGATTCGG	tRF-5c	29	tRNA-Glu-CTC	2.22141851
tRF-1:28-Glu-CTC-1	TCCCTGGTGGTCTAGTGGTTAGGATTCG	tRF-5c	28	tRNA-Glu-CTC	2.17656493
tRF-1:31-Glu-CTC-2	TCCCTGGTGGTCTAGTGGTTAGGATTTGGCG	tRF-5c	31	tRNA-Glu-CTC	2.12466015
tRF-1:28-Gly-GCC-1	GCATGGGTGGTTCAGTGGTAGAATTCTC	tRF-5c	28	tRNA-Gly-GCC	2.12098154
tRF-1:31-Glu-CTC-1	TCCCTGGTGGTCTAGTGGTTAGGATTCGGCG	tRF-5c	31	tRNA-Glu-CTC	2.04337677

## Abbreviations

AH: aqueous humour
AMD: age-related macular degeneration
ANG: angiogenin
ARC: age-related cataract
CNV: choroidal neovascularization
DEG: differentially expressed genes
ERG: electroretinography
HUVEC: human umbilical vein endothelial cells
OCT: optical coherence tomography
RPE: retinal pigment epithelium
tRNA: transfer RNA
tsRNAs: tRNA-derived small non-coding RNAs
tRFs: tRNA derived fragments
tiRNAs: tRNA halves
VASH1: vasohibin 1
