# Supplementary material for: Transfer RNA derived fragment, tRF-Glu-CTC, aggravates the development of neovascular age-related macular degeneration
Source: Theranostics. 2024 Jan 27;14(4):1500–16. doi: 10.7150/thno.92943 (PMC10879880; doi:10.7150/thno.92943)
Supplement: Supplementary file 1 — Supplementary figures and tables. [file thnov14p1500s1.zip › Supplemental Materials.pdf]

Supplemental Information

Transfer RNA derived fragment, tRF-Glu-CTC, aggravates the development of neovascular age-related macular degeneration

Yu Liang, Lingjie Kong, Yuelu Zhang, Yihan Zhang, Mingsu Shi, Jiaqiu Huang, Hongyu Kong, Siyi Qi, Yunlong Yang, Jiaxu Hong, Meidong Zhu, Xiangjia Zhu, Xinghuai Sun, Shujie Zhang, Lianqun Wu, Chen Zhao

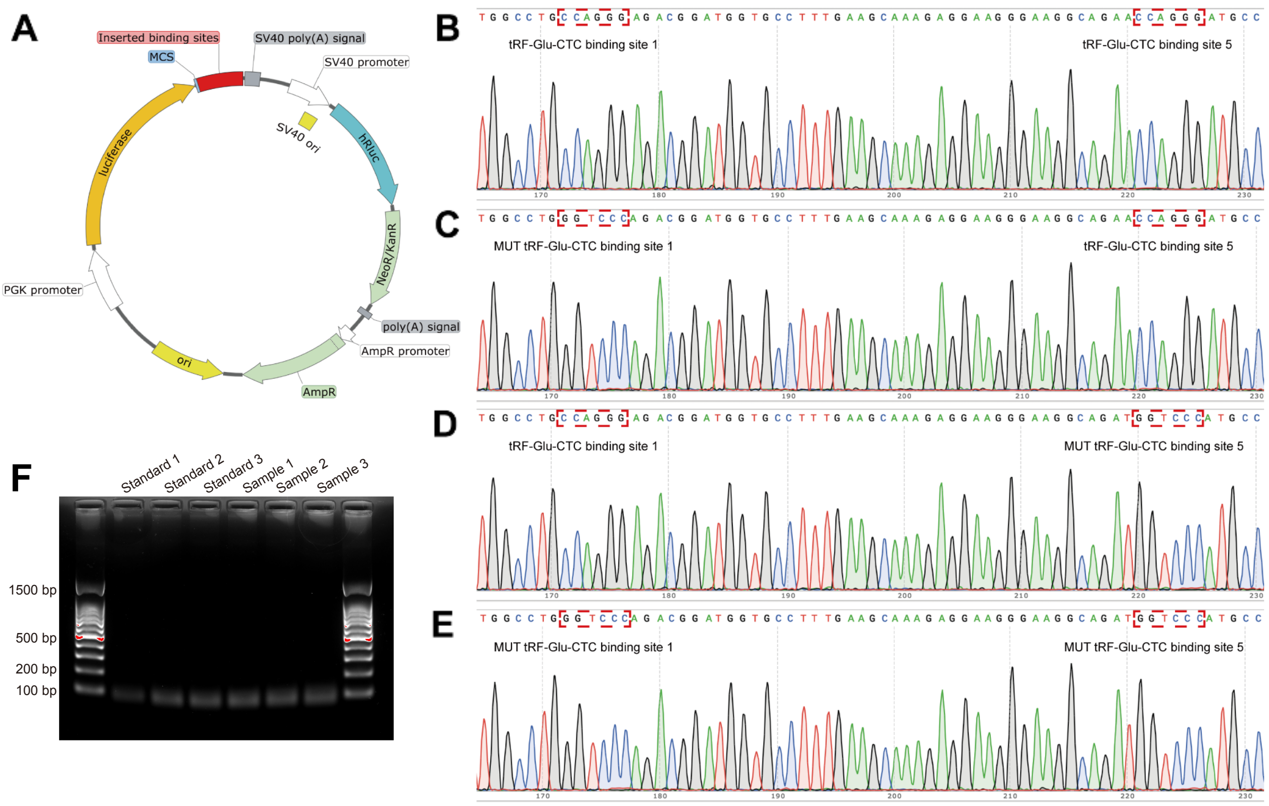

Figure S1. Information about plasmids used in the Dual-luciferase reporter assay. (**A**) The reporter vector used in this study were designed and purchased from Riobio (Shanghai, China). The schematic diagram of the structure of pmirGLO vector and the position of the inserted binding sites. (**B**) The sequences of plasmid containing predicted binding site 1 and site 5 of VASH1 3’UTR region, which was named as pmirGLO-VASH1 WT. (**C**) The sequences of plasmid containing mutant binding site 1 and predicted binding site 5 of VASH1 3’UTR region, which was named as pmirGLO-VASH1 MU1. (**D**) The sequences of plasmid containing predicted binding site 1 and mutant site 5 of VASH1 3’UTR region, which was named as pmirGLO-VASH1 MU2. (**E**) The sequences of plasmid containing mutant binding site 1 and site 5 of VASH1 3’UTR region, which was named as pmirGLO-VASH1 MU3. (**F**) DNA electrophoresis indicated that the sequence length of stem-loop qPCR product from normal samples is consistent with the standard.

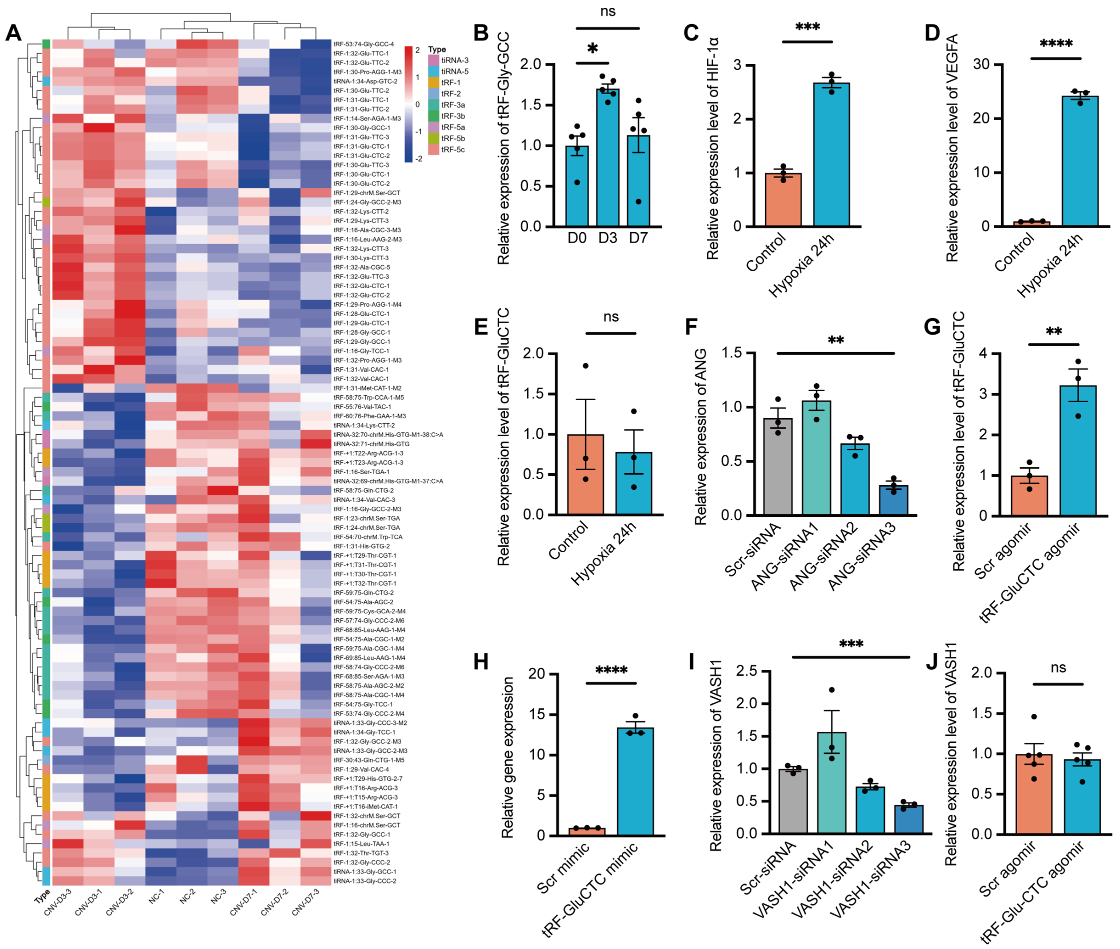

Figure S2. The transfection of siRNA or tRF-Glu-CTC mimic/agomir markedly influenced the expression level of target RNA. (A) Hierarchical cluster heatmap of the top differentially expressed tsRNAs, which were clearly divided into three groups from each other. **(B)** The expression levels of tRF-Gly-GCC at different time points after photocoagulation (n = 5, one-way ANOVA test). (**C**) The expression level of HIF-1α in hypoxia-treated ARPE-19 cell line (n = 3). (**D**) The expression level of VEGFA in hypoxia-treated ARPE-19 (n = 3). (**E**) The expression level of tRF-Glu-CTC in hypoxia-treated APRE-19 (n = 3). (**F**) Compared with ANG-siRNA1 and siRNA2, siRNA3 showed the most significant suppressive effect on the expression of ANG in HUVECs (n = 3, one-way ANOVA test). **(G)** Intravitreal-injected tRF-Glu-CTC agomir enhanced the level of tRF-Glu-CTC in choroid-RPE-sclera complex of mouse (n = 3). **(H)** tRF-Glu-CTC mimic significantly increased the level of tRF-Glu-CTC in HUVECs (n = 3). **(I)** VASH1-siRNA3 significantly reduced the expression of VASH1 in HUVECs (n = 3, one-way ANOVA test). (J) The expression level of VASH1 did not significantly change after the injection of tRF-Glu-CTC agomir (n = 5). ns: not significant, *$P < 0.05$, **$P < 0.01$, ***$P < 0.001$, ****$P < 0.0001$. All data were based

on at least three independent experiments and were demonstrated as the means ± SEM.

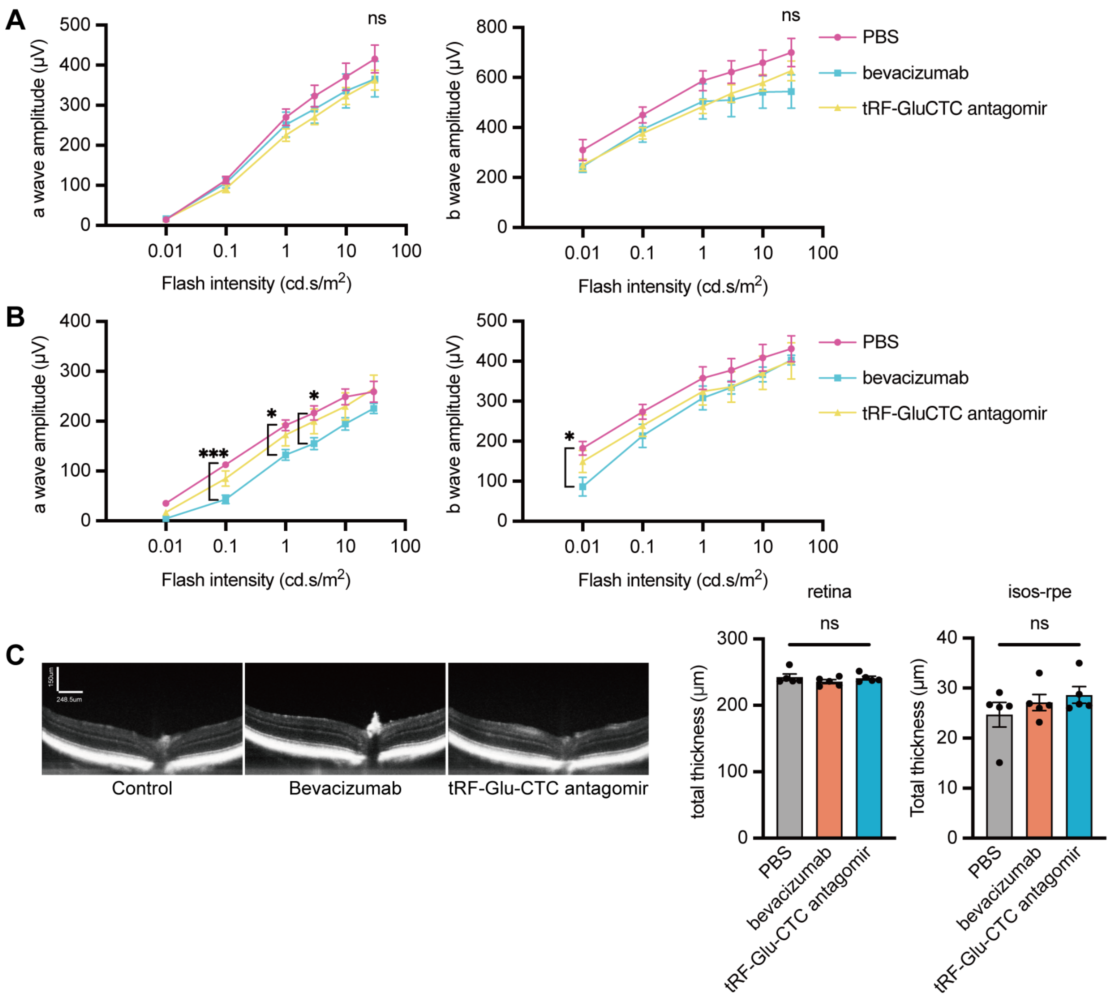

Figure S3. tRF-Glu-CTC antagomir exhibited little neural toxicity in retina of mice. (A-B) The amplitude of the a-wave and b-wave in response to different flash stimuli was measured after 1 week of intravitreal injection (**A**) or after 4 weeks (**B**), showing that there was no difference in neural function between mice injected with PBS or tRF-Glu-CTC antagomir, whereas mice injected with bevacizumab exhibited lower a-wave and b-wave under several stimuli (n = 5, two-way ANOVA test). **(C)** The representative images of OCT images after 4 weeks of weekly PBS, bevacizumab or tRF-Glu-CTC antagomir injections. No alteration of retinal thickness or isos-rpe layer was detected between three groups (n = 5, one-way ANOVA test). *$P < 0.05$, ***$P < 0.001$, ns: not significant. All data were based on five independent experiments and were demonstrated as the means ± SEM.

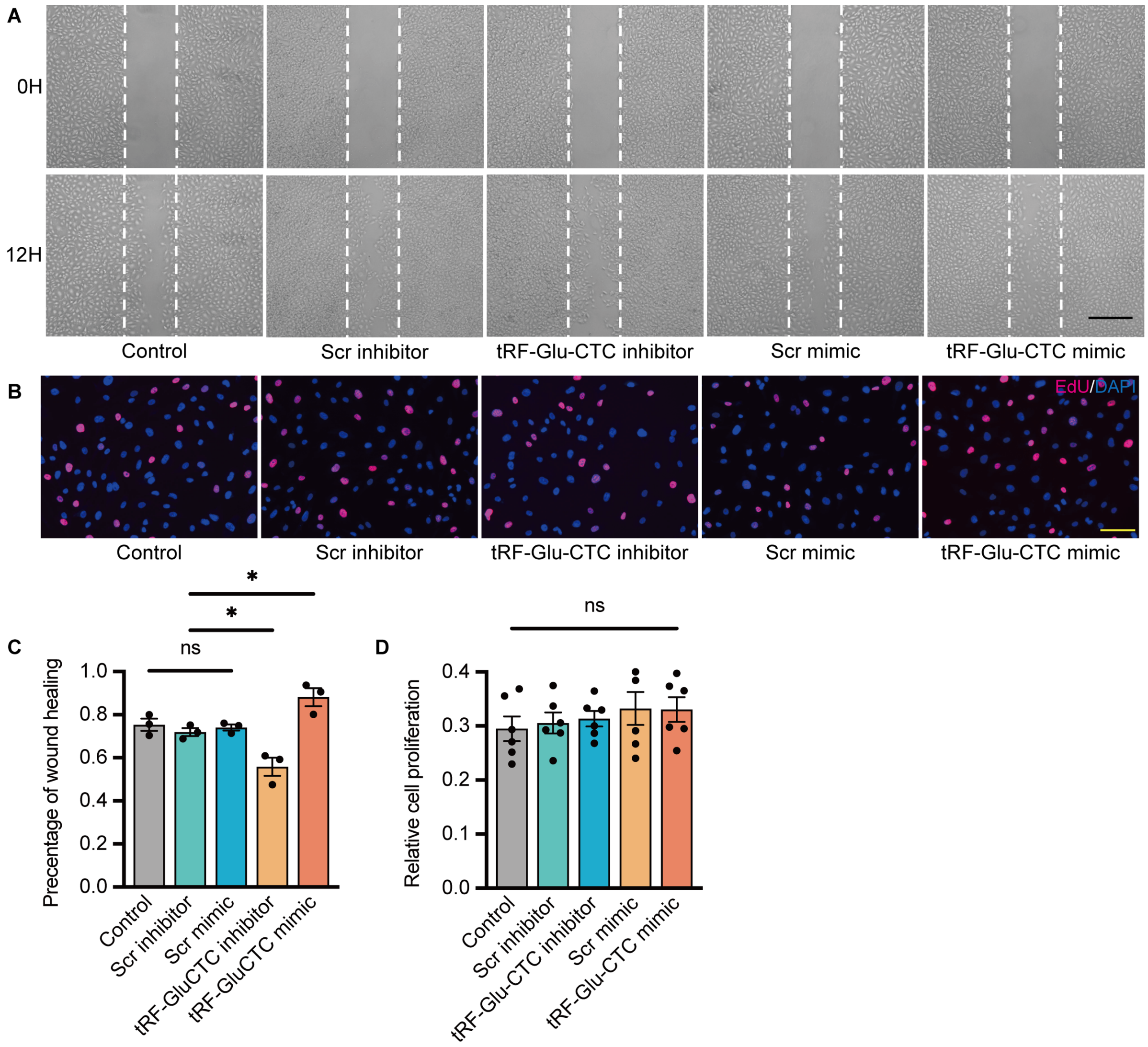

Figure S4. The influence of tRF-Glu-CTC on the migration and proliferation of HUVEC. (A & C) Wound healing assay showed that tRF-Glu-CTC mimic promoted HUVEC migration but did not affect cell proliferation. The remained wound area at 12H was calculated to evaluate the migration ability of HUVECs. (n = 3, one-way ANOVA test). Scale bar, 250 μm. **(B & D)** Edu assay demonstrated that tRF-Glu-CTC mimic or inhibitor had no influence on the proliferation of HUVECs. (n = 6, one-way ANOVA test). *$P < 0.05$, ns: not significant. All data were based on at least three independent experiments and were demonstrated as the means ± SEM.

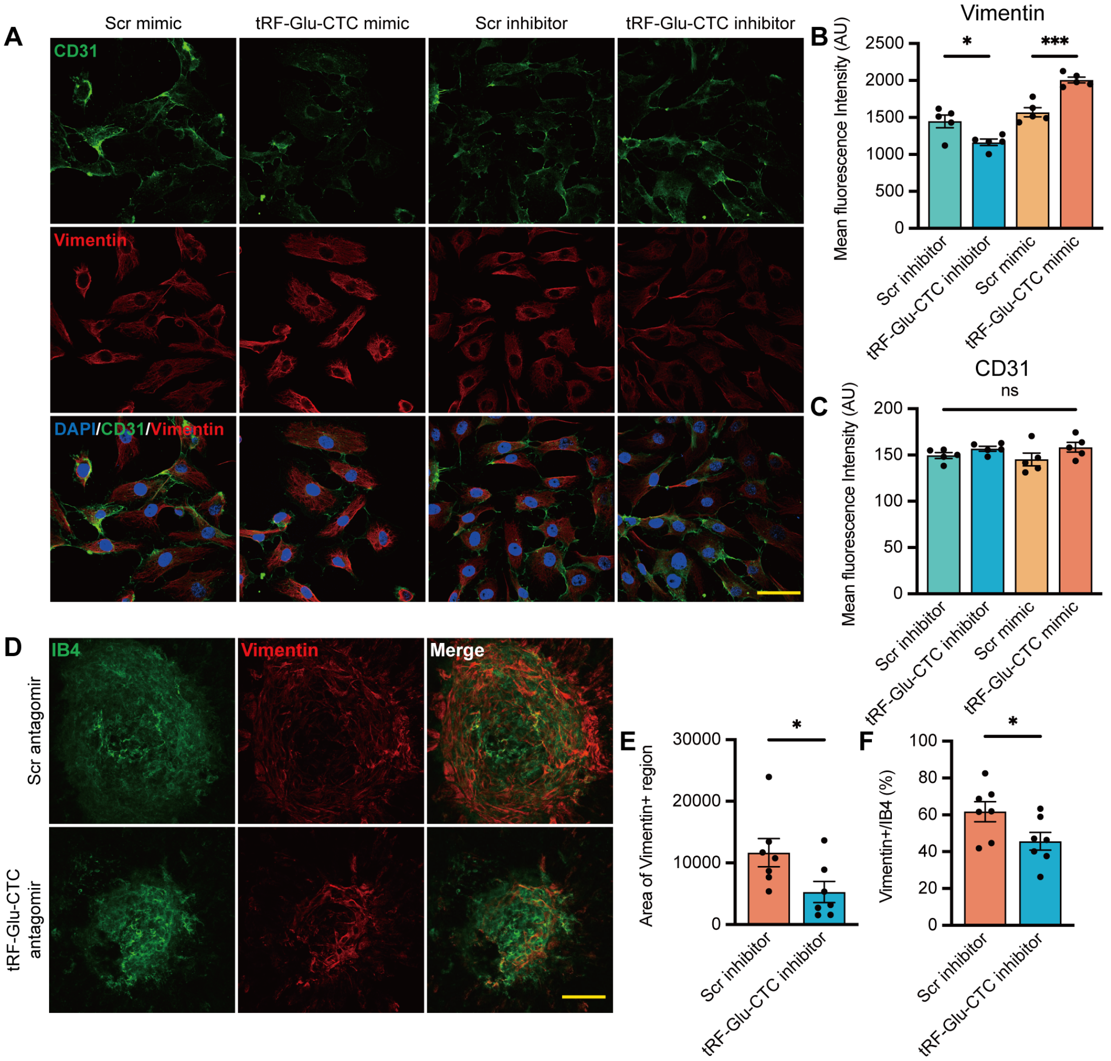

Figure S5. tRF-Glu-CTC regulated the level of vimentin. (A) The immunofluorescence images of HUVECs revealed tRF-Glu-CTC upregulated the expression level of vimentin and decreased CD31 expression. Scale bar, 50 μm. (**B-C)** Mean fluorescence intensity measurement of immunofluorescence images indicated that the level of vimentin was positive correlated with the expression of tRF-Glu-CTC (**B**), while CD31 (**C**) was not (n = 5, one-way ANOVA test). **(D)** Confocal images of choroid flatmounts showed the neovascularization region and vimentin positive region after photocoagulation Scale bar, 50 μm. **(E-F)** The quantification of the area of vimentin positive region (**E**) and the ratio of vimentin+ area/IB4+ area (**F**) showed tRF-Glu-CTC antagomir reduced the vimentin expression in the development of CNV (n = 7). *$P < 0.05$, ***$P < 0.001$, ns: not significant. All data were based on at least five independent experiments and were demonstrated as the means ± SEM.

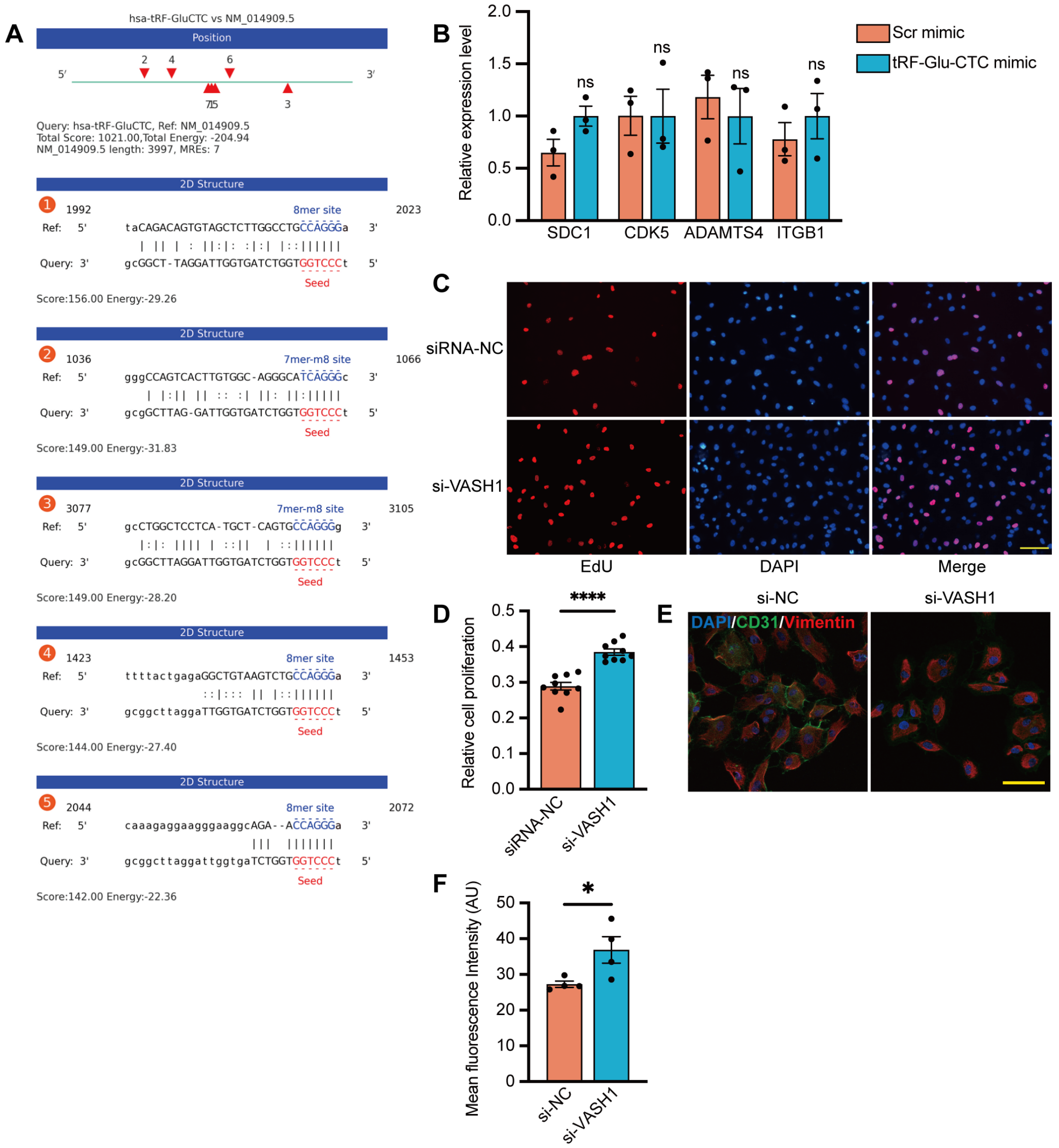

Figure S6. The validation of the function of vasohibin 1 (VASH1) in HUVECs. (A) Five predicted interaction sites between 3’UTR region of VASH1 and tRF-Glu-CTC. **(B)** qPCR assay showed that the expression of other selected genes, including SDC1, CDK5, ADAMTS4 and ITGB1, was not significantly changed by tRF-Glu-CTC mimic. (n = 3) **(C-D)** EdU assay demonstrated that the knockdown of VASH1 significantly promoted the proliferation of HUVECs. Scale bar, 250 μm. (**D**, n = 9). **(E-F)** The expression of vimentin in HUVECs after the down-regulation of VASH1 was presented by immunofluorescence assays, and mean fluorescence intensity measurement indicated that the level of vimentin was increased by si-VASH1 transfection (**F**, n = 4). ns: not significant, *$P < 0.05$, ****$P < 0.0001$. All data were

based on at least three independent experiments and were demonstrated as the means ± SEM.

Table S1. The sequences of oligonucleotides used in the experiment.

Name	sequences (5'-3')
tRF-Glu-CTC mimic	UCCCUGGUGGUCUAGUGGUUAGGAUUCGGCG
tRF-Glu-CTC inhibitor	CGCCGAAUCCUAACCACUAGACCACCAGGGA
tRF-Glu-CTC agomir	UCCCUGGUGGUCUAGUGGUUAGGAUUCGGCG
tRF-Glu-CTC antagomir	CGCCGAAUCCUAACCACUAGACCACCAGGGA
Scr mimic	UUUGUACUACACAAAAGUACUG
Scr inhibitor	CAGUACUUUUGUGUAGUACAAA
Scr agomir	UUUGUACUACACAAAAGUACUG
Scr antagomir	CAGUACUUUUGUGUAGUACAAA
siRNA-NC	UUCUCCGAACGUGUCACGU
ANG-siRNA1	AATGGCTTACCTGTCCACT
ANG-siRNA2	TTTCCGTCGTCCGTAACCA
ANG-siRNA3	GGTTCAGAAACGTTGTTGT
VASH1-siRNA1	CCATCAGCTTCAAGACCTA
VASH1-siRNA2	GCCAATCAAATGCCTGGAA
VASH1-siRNA3	GCGAGGACCTGATGTACAA

Table S2. The information of age-related cataract (ARC) patients and wet age-related macular degeneration (AMD) patients involved in the study.

	ARC (n = 11)	wet AMD (n = 12)	P value
Gender (Female/Male)	7/4	5/7	0.4136
Age, years (mean ± SEM)	68.27±3.171	71.75±3.4	0.4651
Hypertension, No (%)	0	4 (33.3%)	0.0932
Diabetes, No (%)	1 (9.1%)	0	0.4783
Dyslipidaemia, No (%)	1 (9.1%)	2 (16.7%)	>0.9999

Table S3. The primer sequences used in RT-qPCR analysis

primer name	sequence (5'-3')
hsa-ACTB-F	CATGTACGTTGCTATCCAGGC
hsa-ACTB-R	CTCCTTAATGTCACGCACGAT
hsa-VASH1-F	GAAGCCGTGATCCTGGGAATTTACC
hsa-VASH1-R	CTGAGAAGTAGGTCTTGAAGCTGATGG
hsa-ANG-F	CTGGGCGTTTTGTTGTTGGTC
hsa-ANG-R	GGTTTGGCATCATAGTGCTGG
hsa-IL1B-F	ATGATGGCTTATTACAGTGGCAA
hsa-IL1B-R	GTCGGAGATTCGTAGCTGGA
hsa-IL6-F	GACAGCCACTCACCTCTTCAGAAC
hsa-IL6-R	GCCTCTTTGCTGCTTTCACACATG
hsa-IL8-F	CTCTCTTGGCAGCCTTCCTGATTTC
hsa-IL8-R	GGGGTGGAAAGGTTTGGAGTATGTC
hsa-ICAM1-F	GTCACCTATGGCAACGACTCCTTC
hsa-ICAM1-R	AGTGTCTCCTGGCTCTGGTTCC
hsa-SDC1-F	CTGCCGCAAATTGTGGCTAC
hsa-SDC1-R	TGAGCCGGAGAAGTTGTCAGA
hsa-CDK5-F	GGAAGGCACCTACGGAACTG
hsa-CDK5-R	GGCACACCCTCATCATCGT
hsa-ADAMTS4-F	GAGGAGGAGATCGTGTTTCCA
hsa-ADAMTS4-R	CCAGCTCTAGTAGCAGCGTC
hsa-ITGB1-F	CCTACTTCTGCACGATGTGATG
hsa-ITGB1-R	CCTTTGCTACGGTTGGTTACATT
hsa-HIF1A-F	GAACGTCGAAAAGAAAAGTCTCG
hsa-HIF1A-R	CCTTATCAAGATGCGAACTCACA
hsa-VEGFA-F	CTTCTGGGCTGTTCTCGCTTCG
hsa-VEGFA-R	CTCCTCTTCCTTCTCTTCTTCCTCCTC
mmu-ACTB-F	CATTGCTGACAGGATGCAGAAGG
mmu-ACTB-R	TGCTGGAAGGTGGACAGTGAGG
mmu-IL1B-F	TGGACCTTCCAGGATGAGGACA
mmu-IL1B-R	GTTCATCTCGGAGCCTGTAGTG

mmu-IL6-F	TACCACTTCACAAGTCGGAGGC
mmu-IL6-R	CTGCAAGTGCATCATCGTTGTTC
mmu-IL8-F	TGCATGGACAGTCATCCACC
mmu-IL8-R	ATGACAGACCACAGAACGGC
mmu-ICAM1-F	AAACCAGACCCTGGAACTGCAC
mmu-ICAM1-R	GCCTGGCATTTCAGAGTCTGCT
mmu-ANG-F	CTTCGTGCTGGGTCTGGTTGTG
mmu-ANG-R	CTTGGCGTCATGGTGCTGAGTC
mmu-VASH1-F	CACCTGGGAAAGGATGTGGAAG
mmu-VASH1-R	TCGGCTGGAAAGTAGGCACACT

Table S4. The expression pattern of all genes in HUVECs transfected with tRF-Glu-CTC based on RNAseq results.

Table S5. Gene ontology (GO) enrichment analysis revealed enriched terms of differentially expressed genes in HUVECs.

Table S6. Kyoto encyclopedia of genes and genomes (KEGG) pathway analysis revealed enriched pathways of differentially expressed genes in HUVECs.

Table S7. Predicted target genes of tRF-Glu-CTC based on TargetScan and miRanda database.

Table S8. GO enrichment analysis revealed enriched biological process terms of predicted target genes of tRF-Glu-CTC.
